# Survey on the Traditional Use of Medicinal Herbs in Haiti: A Study on Knowledge, Practices, and Efficacy Prevention

**DOI:** 10.3390/plants13172383

**Published:** 2024-08-26

**Authors:** Valendy Thesnor, Yvens Cheremond, Muriel Sylvestre, Patrick Meffre, Gerardo Cebrián-Torrejón, Zohra Benfodda

**Affiliations:** 1 COVACHIM-M2E Laboratory EA 3592, Department of Chemistry, Université des Antilles, UFR SEN, Fouillole Campus, Cedex, 97157 Pointe-a-Pitre, France; tvalendy@gmail.com (V.T.); muriel.sylvestre@univ-antilles.fr (M.S.); 2 URE, Université d’État d’Haïti, Port-au-Prince HT6110, Haiti; cheryvens@yahoo.fr; 3 UPR CHROME, University Nimes, cedex 1, 30021 Nimes, France; patrick.meffre@unimes.fr

**Keywords:** COVID-19, flu, colds, respiratory diseases, TRAMIL

## Abstract

The use of medicinal herbs is highly developed in Haiti. However, there is a significant lack of knowledge in the literature on medicinal plants and their uses. The objective of this study was to determine the knowledge and practices of Haitian families for the prevention/treatment of COVID-19, influenza, and respiratory diseases, as well as the mode of preparation and administration of the plants. Individuals were interviewed using the TRAMIL questionnaire as the information holder. The data obtained were analyzed by calculating 5 indices (relative frequency of citation, use value, the family use value, informant consensus factor, and fidelity level). The study surveyed 120 Haitians and collected 75 plants from 43 botanical families. The botanical family most used for all these preventions and remedies is the Lamiaceae. The highest ranked species with a relative frequency of citation value > 0.3. Infusion, decoction, and in the form of punch are the methods used for the remedies. The study found that the use of herbal remedies is still prevalent in the study area, and many of the commonly used plants have been scientifically validated. However, some plants, such as *Samyda rosea* Sims, lack sufficient research and are recommended for further investigation.

## 1. Introduction

The use of traditional plant-based medicine in Haiti and the Caribbean comes from a combination of African, European, and Indigenous cultural influences, first during the colonial period, which was influenced by the medical conceptions prevailing in Europe from the 16th to the 19th century through the practice of medicine leaves, and second, during the slave period, through African heritage: under the weight of a hard life, the Black slaves tried to cure their ills by resorting to their own knowledge of African medicinal plants for which they found substitutes of American origin or species adapted from their native lands [1]. This knowledge is based on empirical observations but has repeatedly proven its accuracy [2].

In Haiti, the use of medicinal plants is highly developed as the majority of the population uses them, mainly in rural areas but also in the cities [1]. Traditional medicine is often used in Haiti due to its accessibility and affordability compared to conventional drugs, as well as its potential for fewer side effects and a wider range of plant species to choose from for medicinal purposes [1,3]. The use of plants with healing properties is as old as human civilization. According to WHO, in 2008, nearly 80% of the world’s population currently relied on herbal medicines for their primary healthcare needs [4]. Natural products play a dominant role in the discovery of leads for the development of drugs for the treatment of human diseases [5]. Moreover, the effectiveness of medicinal plants used as traditional treatment has been proven by scientific studies [6,7]. Literature data show that several plant extracts, commonly used for the treatment of infectious diseases such as influenza and cold illnesses, demonstrate antiviral, anti-influenza, antibacterial, and anti-inflammatory activities [8,9,10,11,12]. Those infectious diseases are caused by pathogens (bacteria, viruses, parasites, and fungi) and are spread, directly or indirectly, from one person to another [13]. An example of a recent disease is coronavirus 2019 (COVID-19), also known as Coronavirus Disease 2019, a new pandemic resulting from severe acute respiratory syndrome Coronavirus 2 (SARS-CoV-2), which was first reported in late 2019 in Wuhan, China [14,15,16]. Human-to-human contamination is the main mode of transmission of COVID-19 through droplets generated by breathing, sneezing, and coughing [17,18], just like the influenza/cold virus. Other forms of contamination are also possible, such as airborne from infectious aerosols [19,20], contact with an infected surface, and environments in which we live and work [21]. The most common signs and symptoms are fever, cough, myalgia or fatigue and shortness of breath [22], dysfunction of smell and taste [23]. Other symptoms are reported as diarrhea, vomiting, or abdominal pain [24] and also psychological problems such as anxiety and depressive symptoms [25]. COVID-19 has disrupted the lives of many people around the world and has had a huge effect on the health, economy, environment, and social sectors [26]. At this time, there is no specific treatment for the SARS-CoV-2 virus that causes COVID-19.

**Figure 1:**
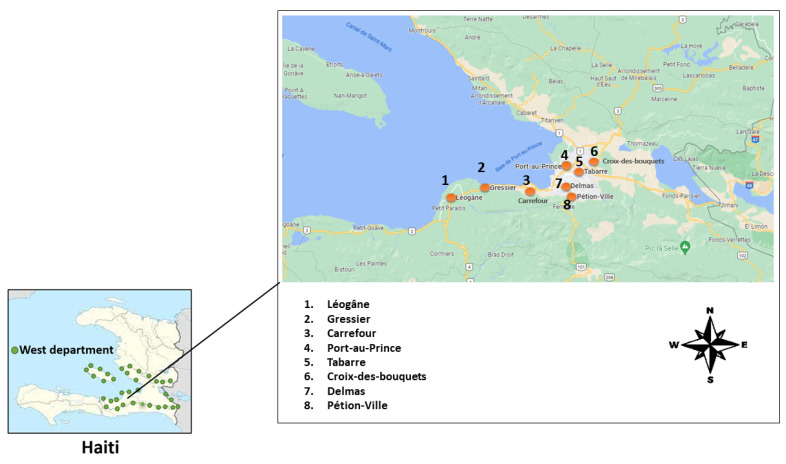
Haiti maps with study areas (Dots correspond to survey locations).

In Haiti, where conventional health systems are not very accessible, treating oneself with plants is the patient’s first resort [3]. Due to the traditional plant knowledge of Haitians, the following study was conducted in the western department of Haiti between July and September 2021 to obtain data on the types of plants used for the treatment and/or prevention of these diseases: influenza, colds, COVID-19, and respiratory diseases (Figure 1). Ethnopharmacological information has an undeniable value of its own as it not only preserves age-old tradition but also effectively guides modern research on bioactive ingredients [27].

In this study, we delved into the realm of Haitian folklore medicine. Using a TRAMIL (Program of Applied Research to Popular Medicine in the Caribbean) inspired methodology, we conducted an ethnobotanical survey through face-to-face interviews and telephone conversations. Our research meticulously documented the local use of medicinal plants in Haiti, underscoring their crucial role in community healthcare. We carried out a comprehensive analysis comparing the use of these plants in treating various infectious diseases. Moreover, we evaluated several ethnobotanical indices to gain a deeper understanding of the cultural and medicinal significance of these plants. This investigation aims to pave the way for the scientific community to explore new antiviral strategies and for the local community to enhance their traditional medicine practices.

## 2. Results

### 2.1. Profile of Medicinal Plant Users

A total of 120 families were interviewed in this study, with one member in each family. Each person in the survey represents a household in general, meaning that several members of the same household are not represented in the survey as multiple different participants, but rather, they are considered to be one respondent for each household. In other words, the survey is designed to capture the opinions and behaviors of households as a unit rather than individual family members separately. Therefore, if multiple members of a household participated in the survey, their responses would be aggregated and considered to be one for that particular household. The representatives of the 120 families were 81.67% (98/120) female and 18.33% (22/120) male. A total of 81% of the respondents were women in our survey because, in Haiti, women appear to be better informed than men [3]. They were all adults between the ages of 18 and 70 years, with an average age of 39 years (Table 1). They were all Haitians living in Haiti. Families living in Delmas represent 35.83% (43/120), those living in Port-au-Prince represent 16.67% (20/120), those living in Pétion-Ville represent 16.67% (20/120), those living in Tabarre represent 8.33% (10/120), those living in Croix-des-Bouquets represent 8.33% (10/120), those living in Léogâne represent 5% (6/120), those living in Gressier represent 5% (6/120) and those living in Carrefour represent 4.17% (5/120).

### 2.2. Plant Families and Remedies

The plants collected represent 75 plants divided into 43 botanical families. The 120 Haitians participants who were interviewed mentioned a total of 149 remedies for flu/cold, respiratory diseases, and COVID-19. Each family participating in the survey could use more than one remedy for a given condition.

**Figure 2:**
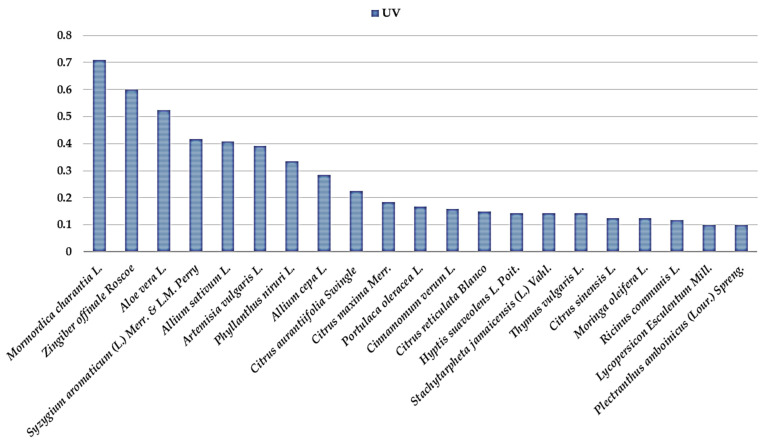
UV value for COVID-19 plants.

**Figure 3:**
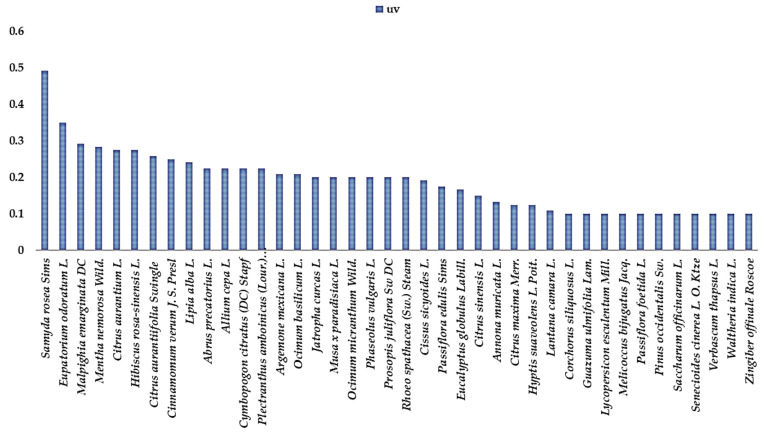
UV value for plants used for flu and colds.

The botanical families most used for all these preventions and remedies are the Lamiaceae (Table 2), with 8 plant species (9.39% were mentioned to fight respiratory diseases, 57.14% flu and cold, and 33.47% to fight COVID-19), the Rutaceae with 5 plant species (8.13% used against respiratory diseases, 51.20% against flu and cold and 40.67% against COVID-19), the Amaryllidaceae with 2 plant species (0.88% used against respiratory diseases, 25.66% against flu and cold and 73.45% against COVID-19), Asteraceae with 3 plant species (5.5% used against respiratory diseases, 49.54% against flu and cold and 44.95% against COVID-19), Zingiberaceae with 2 plant species (10.68% used against respiratory diseases, 11.65% against flu and cold and 77.67% against COVID-19), Myrtaceae with 2 plant species (25% used against respiratory diseases, 21% against flu and cold and 54% against COVID-19) and Curcubitaceae with 1 plant species (7.61% against flu and cold and 92.39% against COVID-19). The highest ranked species with an RFC value > 0.3 (Table 3) is the *Zingiber officinale* Roscoe (0.79), *Mormordica charantia* L. (0.76), *Aloe vera* L. (0.53), *Allium cepa* L. (0.51), *Syzygium aromaticum* L. (0.51), *Samyda rosea* Sims (0.51), *Citrus aurantiifolia* (Christm.) Swingle (0.49), *Allium sativum* L. (0.43), *Eupatorium odoratum* L. (0.42), *Cinnamomum verum* J. S. Presl (0.41), *Citrus aurantium* L. (0.39), *Artemisia vulgaris* L. (0.39), *Phyllanthus niruri* L. (0.33), *Cymbopogon citratus* (DC.) Stapf. (0.32), *Malpighia emarginata* Dc (0.32), *Mentha nemorosa* wild. (0.32) *Citrus sinensis* L. (0.31) and *Citrus maxima* Merr. (0.31). The results showed the ICF values of the 3 conditions mentioned by the families in the study. Flu/cold (0.97) and COVID-19 (0.96) had the highest ICF values, followed by respiratory diseases (0.91). These high ICF values for the different ailments could indicate that they were common, that there is agreement on the need to treat these diseases, and that there is common knowledge among local people using herbal medicines. The highest UV values for each chosen category have functions of a UV value ≥ 0.1. For the plants used against COVID-19 the most important (Figure 2) were *Mormordica charantia* L. (0.71), *Zingiber officinale* Roscoe (0.6), *Aloe vera* L. (0.53), *Syzygium aromaticum* L. (0.42), *Allium sativum* L. (0.41), *Artemisia* (0.39) and *Phyllanthus niruri* L. (0.33). For influenza and cold the most important (Figure 3) were *Samyda rosea* Sims or *Samyda dodecandra* Jacq (0.49), *Eupatorium odoratum* L. (0.35), *Malpighia emarginata* DC. (0.29), *Mentha nemorosa* wild (0.28), *Citrus aurantium* L. (0.28), *Hibiscus rosa-sinensis* L. (0.28), and *Citrus aurantiifolia* (Christm.) Swingle (0.26) and for respiratory diseases the most important were *Eucalyptus globulus* Labill. (0.13) and *Ricinus communis* L. (1.13).

### 2.3. Plants, Remedies, and Methods of Administration

A total of 149 remedies were reported for 75 plants, with 16 used for respiratory diseases, 53 for COVID-19, and 55 for flu and/or colds. Traditional preparations included decoctions and infusions (63.33%), punch (16%), inhalation (9.67%), fruit/leaf juice (8.67%), and baths and massage (2.33%).

Infusion is the most preferable method of preparation for the softer parts, such as flowers and leaves, because the plant part does not boil with water [28]. Examples of flu remedies are the flowers and leaves of *Moringa oleifera* L. and the flowers of *Hibiscus rosa-sinensis* L., the latter given mainly to children by most of the families interviewed. The harder parts, such as roots, rhizomes, underground parts, seeds, and fruits, are proposed to be prepared in decoction because boiling with water extracts the maximum of bioactive compounds. We have as an example the *Cymbopogon citratus* (DC) Stapf. that is used in decoction form (root and leaf). When it is with the leaves only, the decoction time is minimum specified by the informants and can also be used in infusion.

Punch is a commonly used preparation method for COVID-19 remedies (Table 4) [33], which consists of a mixture of up to 6 plants believed to help fight against symptoms and complications. Certain punch remedies may incorporate protein-rich ingredients like milk and eggs, which can fortify the immune system and provide essential strength, while others may feature the addition of rum [33]. Proteins, being essential macronutrients, play a crucial role in antibody production and can aid in the fight against COVID-19 [34]. Inhalation of hot steam with plants like *Eucalyptus globulus* Labill, *Syzygium aromaticum* L., *Zingiber officinale* Roscoe, or the effects of oils like *Ricinus communis*, is another method that has been used for specific plants. This method is believed to help thin mucus and fight against microbes in the throat or nose [28,29,33]. For fruit/leaf and/or edible juices, families consume more fruit and vegetables to strengthen their immune systems. Also, there are plants like *Eupatorium odoratum* L. and *Allium cepa* L., or they can rub/blend to take a spoonful of juice against flu [28,33]. For baths and/or massages [28,29,33], we have, for example, the oil of *Ricinus communis* L., which is used to massage the body that has aches or to massage the stomach in the case of strong flu or the leaves for baths with other plants like *Mormordica charantia* L. and *Ocimum basilicum* L.

Therefore, for all the remedies quoted in the survey, 88% are administered orally and recommended 1 to 2 times a day for prevention against COVID-19 and up to 3 times a day for people with COVID-19. For punches (Table 4) that have both medicinal and aromatic plants with protein additives or rum it is recommended once a day. In Haiti before COVID-19, people with flu/cold and respiratory diseases used to take the remedies up to 2 times a day [28,29]. For some remedies COVID-19, unlike other teas that families took in the form of a mixture before COVID-19 for flu/cold, there are more herbs added in the decoctions COVID-19, which are aimed at combating flu/cold, fever, and pain associated with the disease [33]. For contraindications most of the complex punch remedies are not recommended for children and pregnant or nursing women. In the worst case, they are advised only once a day for those who are severely affected at a lower dose. For decoctions and/or infusions, there was only mugwort (*Artemisia vulgaris* L.) that some families have drawn attention to the toxicity, and as a solution, there are some families that use it more or put a small amount (a small branch) in 1 to 2 L of water.

### 2.4. Plants and Fidelity Levels

The level of fidelity (FL) of a given plant for a given use was calculated to rank the recorded plant species according to their claimed relative efficacy. Species with RFCs less than 0.1 (Table 2) were not included in the FL calculations (Table 5). Table 5 shows the FL% value for the efficacy of a certain plant to treat a certain disease (Table 6) or to be designated for primary use.

For the treatment of influenza, the species recorded at 100% FL were *Abrus precatorius* L., *Argemone mexicana* L., *Cissus sicyoides* L., *Corchorus siliquosus* L., *Guazuma ulmifolia* Lam., *Lantana camara* L., *Musa x paradisiaca* L., *Passiflora foetida* L., *Prosopis juliflora* Sw DC, *Rhoeo spathacea* (Sw.) Steam., *Saccharum officinarum* L., *Senecioides cinerea* L. O. Ktze, *Verbascum thapsus* L., *Waltheria indica* L. followed by *Samyda rosea* Sims (96.72%), *Malpighia emarginata* DC (92.11%), *Mentha nemorosa* wild. (89.47%), *Ocimum micranthum* wild. (82.76%), *Lipia alba* L. (74.36%), *Phaseolus vulgaris* L. (70.59%), *Melicoccus bijugatus* Jacq (75%), *Cymbopogon citratus* (DC) Stapf (71.05%), *Plectranthus amboinicus* (Lour.) Spreng (69.23%), *Ocimum basilicum* L. (64.1%), *Myristica fragrans* L. (61.54%), *Mammea americana* L. (61.11%), *Annona muricata* L. (57.14%), *Cinnamomum verum* J. S. Presl (61.22%), *Citrus aurantiifolia* (Christm.) Swingle (52.54%), *Citrus aurantium* L. (70.21%) and *Eucalyptus globulus* Labill. (51.28%). For COVID-19 treatments, the species obtained at 100% fidelity level were *Aloe vera* L., *Phyllanthus niruri* L., *Portulaca oleracea* L., *Artemisia vulgaris* L et *Stachytarpheta jamaicensis* (L.) Vahl., followed by *Allium sativum* L. (94.23%), *Mormordica charantia* L. (92.39%), *Syzygium aromaticum* L. (81.97%), *Zingiber officinale* Roscoe (75.79%), *Thymus vulgaris* L. (68%), *Citrus reticulata* Blanco (64.29%), *Moringa oleifera* L. (60%), *Allium cepa* L. (55.74%), *Citrus maxima* Merr. (59.46%) and *Hyptis suaveolens* L. Poit. (53.13%).

## 3. Discussion

This study documents the traditional medicinal plants used for the prevention and/or treatment of influenza, respiratory diseases, and COVID-19 in eight communes of the West Department in Haiti. Using the TRAMIL method, herbal remedies identified in the survey were included in the data when the calculated frequency (Freq = Ni/Np × 100) was 20% or higher [94]. Also, a minimum value of five was set for the values Ni and Np. The TRAMIL group chose this minimum frequency to exclude anecdotal uses. Plants commonly used to treat various conditions, such as *Mormordica charantia* L., *Zingiber officinale* Roscoe, *Aloe vera* L., and *Eupatorium odoratum* L., have been found to possess antimicrobial properties that can help the body fight against bacterial and viral infections [10,57,95,96]. The extract of *Mormordica charantia* L. in Trinidad and Tobago has demonstrated strong potential in inhibiting multi-resistant clinical bacterial isolates [95]. The presence of kaempferol and quercetin derivatives, including rutin, in the extract, suggests that these compounds may be responsible for the observed activity [95]. *Aloe vera* L. in Iran has been clinically proven to reduce the frequency of infections and fever and increase the count of white and red blood cells in 60 children who have acute lymphocytic leukemia. In the survey, the 120 informants used different parts of the plants [97]. Different parts of plants produce chemicals that are known as bioactive secondary metabolites [57]. These metabolites include alkaloids, quinines, flavones, lectins, polypeptides, flavonols, flavonoids, coumarin, tannins, terpenoids, essential oils, etc. These compounds are available and can be derived from leaves, bark, roots, flowers, fruits, and seeds [38,57,98]. Regarding the pathologies, the remedies for the prevention and treatment of COVID-19 are the most cited. For not only did Haitians use remedies with one herb but also with several herbs as a mixture as they targeted almost all symptoms related to COVID-19, such as: cough, respiratory problems, fever, gastrointestinal problems, pain, etc. As recently reported by the authors [99], herbal mixtures reduce hospitalization rates and time to recovery from symptoms. Most recipes involved decoctions. Administration was either oral or applied topically for soreness. Several plants that were frequently mentioned in the survey, such as *Zingiber officinale* Roscoe, *Mormordica charantia* L., *Aloe vera* L., *Allium cepa* L., *Allium sativum* L., *Syzygium aromaticum* L, *Citrus aurantiifolia* (Christm.) Swingle, *Eupatorium odoratum* L., *Cinnamomum verum* J. S. Presl, *Citrus aurantium* L., *Artemisia vulgaris* L., and *Phyllanthus niruri* L., have been validated through pharmacological studies for their antiviral, antibacterial, or pain management properties. These plants were found to be potential remedies for COVID-19 in the study, as they were mentioned frequently with an RFC value higher than 0.3. However, there is limited information available regarding the pharmacological properties of *Samyda rosea* Sims (also known as *Samyda dodecandra* Jack.), which was also frequently cited. This plant is naturalized in Haiti and can be found in other Caribbean regions like Cuba, Bonaire, and Curacao [100]. Haitians have been using it mainly against flu for many years (FL = 96.72%), but there is no literature available regarding its use elsewhere. For the other most cited plants in the survey, despite their frequent use in natural medicine, the Caribbean wild variety is poorly studied for pharmacological activities related to COVID-19 and its corresponding symptoms.

*Zingiber officinale* Roscoe (Ginger) aromatic herb with leafy stem and horizontal rhizomes, native to and naturalized in India, was the most frequently cited herb in the remedies; it was described as being used mainly against COVID-19 (FL = 75.789%) in a study the authors showed that ginger compounds, specifically 6-gingerol, 8-gingerol, and 6-shogaol, can induce rapid relaxation in human airway smooth muscle and attenuate airway resistance in mice [101]. Recently, another study showed that 6-gingerol, a compound found in *Z. officinale* (ginger), has significant anti-influenza activity against the PR8 strain of the H1N1 influenza virus, with an IC50 value of 2.25 ± 0.18 μM [102]. In Morocco, it is traditionally used for gastrointestinal disorders (FL = 86%) [103] and The administration of an acetone extract (at 1000 mg/kg), zingiberene (at 100 mg/kg, the main terpenoid of the extract), and 6-gingerol (at 100 mg/kg) resulted in significant inhibition of HCl/ethanol-induced gastric lesions, with reductions of 98%, 54%, and 54%, respectively [10,104]. The hydroethanol extract of ginger shows antibiotic activity against Gram-positive and Gram-negative bacteria [10,105]. 10-Gingerol and 6-gingerdiol had potent antifungal activity against 13 human pathogens at concentrations below 1 mg/mL [106]. Treatment of ginger root with boiling water abolished the antibacterial effect against *E. coli*, *Salmonella typhimurium*, *Vibrio parahaemolyticus*, *Pseudomonas aeruginosa*, *Proteus vulgaris*, *Staphylococcus aureus*, *Mycobacterium phlei*, *Streptococcus faecalis* and *Bacillus cereus* except that against the growth of Micrococcus luteus. This indicates that the antibacterial principle is heat labile [10,107]. It has been shown that fresh ginger could prevent human respiratory syncytial virus (HRSV) infection largely by inhibiting viral attachment [9]. This may partly explain the common use of *Zingiber officinale* Roscoe against COVID-19 with associated symptoms.

*Mormordica charantia* L is a plant that originated from Asia and has been naturalized in the Caribbean [28,29]. Although it is a climbing plant that can reach 3 to 4 m in height [28], in Haiti, it is traditionally used mainly to combat fever (FL = 92.391%) as a possible treatment against COVID-19. The fleshy part and seeds of the plant are also a good source of phenolic compounds, such as catechin, epicatechin, and gallic acid [108]. The plant is used in traditional medicine as an antidiabetic, anthelmintic, laxative, or aphrodisiac [12,108]. The different parts of the plant are also used to treat ailments related to colic, diarrhea, eczema, fever, hemorrhoids, inflammation, malaria, dysentery, sores, anemia, cough, scabies, asthma, wounds, and infections [12]. The protein purified from Bitter Gourd seed possessed effective antiviral activity against a wide range of influenza A subtypes, including H1N1, H3N2, and H5N1 [11]. Another study conducted in Trinidad and Tobago found that the plant extract contains phenolic acid derivatives and flavonoid glycosides that have strong antimicrobial potential against multi-resistant clinical bacterial isolates, likely due to the presence of flavonoids such as quercetin, kaempferol, quercitrin, and rutin [95]. These reports therefore correlate with the traditional uses of *Mormordica charantia* L.

*Aloe vera* L. (Aloe) is an ornamental and medicinal plant native to North Africa. Aloe is used in traditional medicine and the treatment of burns and wounds. It has various therapeutic properties such as antiviral, antidiabetic, laxative, protective against radiation, antiallergic, anti-inflammatory, anticancer and immunostimulant, antibacterial, anti-oxidant, and photoprotective activities [39,96]. In Haiti, it was described as being used mainly against COVID-19 (FL = 100%). Recently, researchers have assessed the antibacterial, anti-oxidant, photoprotective, and cytotoxic activities of extracts from *Aloe vera* L. The results indicated that the n-butanol fraction, as well as the acetone and methanol extracts, demonstrated antibacterial effects against a variety of bacterial strains, including *S. aureus*, *B. cereus*, *E. coli*, *P. aeruginosa*, and *A. baumanii*, with MIC values ranging from 1.25 to 10 mg/mL [96]. Another study aimed to evaluate the antibacterial potential of methanol and acetone extracts of aloe vera against various bacterial strains [109]. The findings revealed that the methanol extract exhibited the highest antibacterial activity against B. *cereus*, with a zone of inhibition of 22.33 mm, while the acetone extract exhibited lower values of zones of inhibition, ranging from 6.00 mm for *E. coli* to 7.33 mm for *S. pyogenes* [109]. The extracts showed greater antibacterial activity against Gram-positive bacteria compared to Gram-negative bacteria. Moreover, the study authors identified Pyrocatechol, Cinnamic acid, p-coumaric acid, and Ascorbic acid as the active compounds responsible for the maximum antibacterial activity [109].

*Allium cepa* L. (onion) is a perennial herbaceous plant characterized by its bulb. It is native to Asia and cultivated in various countries. In this survey, almost all families using onion in remedies specified red onions, which they use for COVID-19 with symptoms such as cough and respiratory problems (FL = 55.737%). However, according to the literature, red onions have more pronounced anti-oxidant activities than white onions [109]. Moreover, it has been demonstrated that red onions can increase the rate of respiration [110], which could be beneficial against respiratory viruses such as COVID-19. In fact, it has been suggested that breathing in the odor emanating from a raw cut onion for a few minutes could be helpful against such viruses [110]. In another study, four polysaccharide fractions were extracted from onion and tested for their antibacterial activity against Gram-positive bacteria (*S. aureus*, *B. subtilis*) and Gram-negative bacteria (*E. coli*, *S. typhimurium*). The results showed that all four polysaccharide extracts inhibited the growth of both types of bacteria [111].

*Allium sativum* L. (Garlic) is an annual bulbous herb that originates from Central and South Asia and is widely cultivated in warm and dry climates worldwide [112]. In this survey, Haitians reported using garlic with an FL of 94.23% and a UV of 0.41 to prevent COVID-19 and its various symptoms. In contrast, a study from Morocco reported a lower FL of 64% and a UV of 0.10 for the use of garlic in microbial infections [103]. Garlic is commonly used in food and traditional medicine to treat various ailments, including colds, fever, coughs, asthma, and wounds, and as an antimicrobial agent [38,112]. A study has identified organosulfur compounds, such as allicin and its derivatives (ajoene, allitridin, and garlicin), as the most promising compounds responsible for garlic’s therapeutic activities, especially in the prevention of viral infections [112]. Garlic has been used in African traditional medicine, such as in Ethiopia and Nigeria, to treat various infections, including sexually transmitted diseases, tuberculosis, respiratory tract infections, and wounds [112]. Allicin has been identified as one of the main organosulfur compounds responsible for antiviral activity [113]. In vitro and in vivo studies have demonstrated the antiviral potential of garlic and its organosulfur compounds against a wide range of viruses [112].

*Syzygium aromaticum,* also known as clove, is native to Indonesia but is now cultivated in many parts of the world [90]. In our study, clove was reported as a COVID-19 remedy with a frequency of 81.967%. According to a study, the aqueous extract of clove seeds showed antimicrobial activity against Escherichia coli, Pseudomonas aeruginosa, and Staphylococcus aureus with minimum inhibitory concentration (MIC) and minimum bactericidal concentration (MBC) values of 0.06 and 0.10 mg/mL, respectively [90]. Another study found that the MeOH crude extract of *Syzygium aromaticum* inhibited the growth of Gram-negative anaerobic periodontal pathogens, including *Porphyromonas gingivalis* and *Prevotella intermedia* [114]. Eight active compounds were identified, including 5,7-dihydroxy-2-methylchromone 8-C-β-d-glucopyranoside, biflorin, kaempferol, rhamnocitrin, myricetin, gallic acid, ellagic acid and oleanolic acid [114]. These findings suggest that clove has potential antimicrobial properties that could be useful in the treatment of COVID-19 and other infections.

*Citrus aurantiifolia* (Christm.) Swingle (Lemon) is a shrubby tree widely cultivated in warm subtropical and tropical regions of the world. The Haitians use the native lemon mainly for flu (FL = 52.542). In the literature, lemon oil has antimicrobial, fungicidal, insecticidal, and anticancer properties [31]. In a study, the results showed promising in vitro anti-oxidant and cytotoxic activities, especially for the essential oil of the leaves [31]. The essential oil of *Citrus aurantifolia* (Christm.) Swingle was extracted using hydrodistillation, and its antimicrobial activity was assessed against a representative range of Gram-positive bacteria, Gram-negative bacteria, and yeasts, including both ATCC and clinical strains. The disc diffusion and broth microdilution methods were used for evaluation. The major components of the lime essential oil were found to be β-pinene (12.6%), limonene (53.8%), γ-terpinene (16.5%), terpinolene (0.6%), α-terpineol (0.4%), and citral (2.5%). These compounds are likely responsible for the oil’s good antimicrobial activity, particularly against Gram-positive bacteria such as *Staphylococcus aureus*, *Bacillus subtilis*, and *Staphylococcus epidermidis* [115].

*Eupatorium odoratum* L. (Siam weed). A study performed a phytochemical analysis on siam weed to study the anti-oxidant and antimicrobial activities of ethanolic and aqueous extracts of its leaves [57]. The authors show that the ethanolic extract of leaves showed anti-oxidant activity and antibacterial properties superior to those of the aqueous extract [57]. This may explain the use of this plant by the Haitians for flu (FL = 84%). The study examined the antibacterial activity of *Eupatorium odoratum* L leaf extracts against several bacterial strains, including *Klebsiella oxytoca*, *Salmonella enterica*, *Shigella sonnei*, and *Vibrio cholerae*. The results showed that the extracts displayed antibacterial activity within the range of 0.156 to 1.25 mg/mL. Among the strains tested, *V. cholerae* exhibited the highest susceptibility to the extracts, with a minimum inhibitory concentration (MIC) of 0.156 mg/mL for the dichloromethane extract and 0.312 mg/mL for the butanol extract. The study also identified sinensetin and scutellareintetramethyl ether as the active components responsible for the antibacterial properties of *Eupatorium odoratum* L. [116].

*Cinnamomum verum* J. S. Presl. (Cinnamon) native to Sri Lanka and southern India but also distributed in Southeast Asia, China, Burma, Indonesia, Madagascar, the Caribbean, Australia, and Africa. In a study, authors show the antibacterial effect of methanol, ethanol, petroleum ether, and ethyl acetate extracts of *C. verum* against *Staphylococcus aureus*, *Bacillus subtilis*, *Escherichia coli*, and *Pseudomonas aeruginosa*. Ethyl acetate extract was reported to possess the strongest antibacterial activity against *Staphylococcus aureus*, *Escherichia coli*, and *Pseudomonas aeruginosa*, while Bacillus subtilis had shown the maximum sensitivity towards petroleum ether extract [30]. In another study, the chemical composition of cinnamon bark extract was analyzed, revealing cinnamal and coumarin as the main compounds [117]. The extract showed antimicrobial activity against various pathogenic and spoilage microorganisms, including *Staphylococcus aureus, Staphylococcus epidermidis, Salmonella abony, Escherichia coli, and Candida albicans,* except for some strains of *Pseudomonas* species [117]. It also reduces the metabolism of bacterial cells, coagulates cellular materials, and limits the rate of replication [30,118]. These studies can explain the use of this plant by the Haitians for flu FL = 61.22%.

*Citrus aurantium* L. is used in traditional medicine in India for treating stomach aches, vomiting, high blood pressure, dysentery, diarrhea, cardiovascular analeptic, as a sedative, for boils, and for urinary tract infections. Its essential oil from fruit peels has anti-oxidant, antimicrobial, antifungal, antiparasitic, and anti-inflammatory activities [32]. In our study, Haitians use *Citrus aurantium* L. mainly for flu (FL = 70.21%). In a study the auteurs show essential oil extracted from leaves exhibited strong antifungal activity against two strains of *Candida albicans*, with fungicidal effect. The MIC of essential oil against *Candida albicans* strains was 0.15–0.31% (*v*/*v*) [32]. Another study investigated the antibacterial properties of citrus oil and identified d-limonene as its primary chemical constituent [119]. The results showed that the essential oil exerted dose-dependent antibacterial activity, with a greater impact on *S. aureus* (5.8–7.9 mm) compared to *E. coli* (1.7–3.1 mm), as demonstrated by the diameter of the zone of inhibition [119].

*Artemisia vulgaris* L. (mugwort) is one of the important medicinal plant species of the genus Artemisia and probably the most common in Haiti today. This medicinal plant has a wide spectrum of therapeutic properties, among which are antimalarial, anti-inflammatory, antihypertensive, anti-oxidant, antitumor, immunomodulatory, hepatoprotective, antispasmodic and antiseptic [44]. The antimicrobial activity of mugwort oil was studied against *Staphylococcus aureus*, *Staphylococcus epidermidis*, *Enterococcus faecalis*, *Enterobacter cloacae*, *Escherichia coli*, *Salmonella typhimurium*, and *Candida albicans* using the disc diffusion method in comparison with the positive control (ceftazidime and ketoconazole) [44,120]. In a study, the antibacterial activity of the methanol extract and essential oil of *A. vulgaris* was investigated, with both showing significant activity against the test organism [121]. The methanol extract exhibited higher activity against *Bacillus subtilis* and *Enterococcus* spp., with zones of inhibition of 12.48 mm and 12.06 mm, respectively [121]. The essential oil of *A. vulgaris* was found to contain mono- and sesquiterpenes, including sabinene, β-thujone, chrysanthenone, camphor, borneol, and germacrene [121]. In our study, Haitian use mugwort only for COVID-19, and they mentioned in the survey that they need a very small quantity for the infusions, and this corresponds with these studies [122,123]. It is reported to become toxic at high or repeated doses, as is the case with some other Artemisia species.

*Phyllanthus niruri* L. this plant is also commonly used to treat hypertension, diabetes, and intestinal infections. It also has a lipid-lowering effect and hepatoprotective activity [98]. In a study, the methanolic extract of *Phyllanthus niruri* L. demonstrated antibacterial activities against all the Gram-positive and Gram-negative was investigated against pathogenic bacteria responsible for common infections of the skin and urinary and gastrointestinal tracts [124]. In this survey, Haitian use it for COVID-19.

The plants described above, including *Zingiber officinale* Roscoe, *Mormordica charantia* L., *Aloe vera* L., *Allium cepa* L., *Allium sativum* L., *Syzygium aromaticum* L., *Citrus aurantiifolia* (Christm.) Swingle, *Eupatorium odoratum* L., *Cinnamomum verum* J. S. Presl, *Citrus aurantium* L., *Artemisia vulgaris* L., and *Phyllanthus niruri* L. contain active ingredients that can be found in the recipes for herbal remedies for COVID-19 and symptoms [37,56,57,125]. These substances can act against flu, fever, and pain. Other secondary substances, such as flavonoids, anthranoids, cyanogenic glycosides, and alkaloids, can act on many symptoms of covid. To take only the example of flavonoids here, let’s say that quercetin, rutin, naringin, and apigenin, found in several species used in recipes, are recognized and sought after for their antiviral, antibacterial, and anti-inflammatory properties. A study using computational analysis has suggested that various flavonoids, such as kaempferol, quercetin, luteolin-7-glucoside, apigenin-7-glucoside, naringenin, catechin, and epigallocatechin, may hold significance as potential inhibitors of the SARS-CoV-2 M^pro^ [126]. Another study found that quercetin may be effective in treating early-stage COVID-19. The quercetin group recovered faster and had more negative SARS-CoV-2 tests than the control group [127].

Studies suggest that environmental factors like temperature, humidity, and air pollution may affect COVID-19 transmission [17,18,19,20,21,128,129]. No specific drug has been validated for COVID-19 treatment in large-scale studies, but remdesivir shows promise as an antiviral. Other treatments, such as umifenovir combined with lopinavir/ritonavir and hydroxychloroquine with azithromycin, have shown some efficacy. Research on teicoplanin and monoclonal and polyclonal antibodies is ongoing. The pandemic has significantly increased deaths, particularly among those with comorbidities like hypertension, obesity, cardiovascular issues, diabetes, and respiratory disorders, overwhelming healthcare systems [128]. Rapid identification and risk stratification of high-risk patients, along with appropriate resource allocation, are crucial for healthcare systems globally.

In response to the pandemic, Haitians have extensively used herbal remedies for COVID-19 prevention and treatment, involving mixtures of herbs targeting symptoms like cough, respiratory issues, fever, gastrointestinal problems, and pain. Typically, these remedies are decoctions, infusions, or punches, often including 6 to 7 plant extracts with ingredients like milk, eggs, and honey. Similarly, traditional Chinese medicines have been used as preventative measures based on past use against H1N1 and SARS, but clinical data on their effectiveness for COVID-19 is lacking [128,130]. Six traditional Chinese medicines were notably prevalent, requiring rigorous clinical studies for validation. Additionally, enhancing immunity with vitamins (A, D, and E) and minerals has been suggested as a preventative measure [129]. Therefore, even if the treatment is successful, it is difficult to indicate an active and beneficial component. Further studies are needed to protect high-risk groups and to investigate the efficacy of herbal mixtures. In the research for new antibacterial, antiviral, and even anti-analgesic molecules with new modes of action, it will be interesting to identify the compounds responsible for the activities and thus to discover new mechanisms to relieve these ailments.

## 4. Materials and Methods

### 4.1. Study Areas

This ethnopharmacological survey was conducted in 8 communes in Haiti between July and September 2021, such as: Port-au-Prince, (18°32′24″ N, 72°20′24″ W), Carrefour (18°32′ N, 72°24′ W), Gressier (18°27′ N, 72°17′ W), Léogâne (18°30′39″ N, 72°38′02″ W), Pétion-Ville (18°31′ N, 72°17′ W), Delmas (18°33′ N, 72°18′ W), Tabarre (18°35′ N, 72°16′ W), and Croix-des-Bouquets (18°35′ N, 72°14′ W) (Figure 1).

### 4.2. Data Collection

The interviews were conducted in Haitian Creole (native language) in the field and also by telephone for certain areas: Carrefour, Gressier, Léogâne and Croix-des-Bouquets. They were a total of 120 people with one person per family. Three of these people were “machann fèy” or, in English, medicinal plant traders, and the others were people who had acquired knowledge of the use of medicinal plants from their parents or relatives.

**Figure 4:**
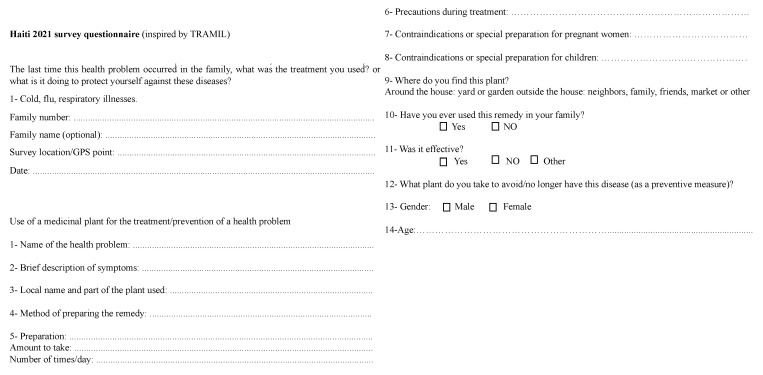
Survey questions inspired by TRAMIL.

The interview methodology (Figure 4) adopted for this survey was inspired by that used by TRAMIL (TRAditional Medicine in the Islands) which is an applied research program on the popular use of medicinal plants in the Caribbean. TRAMIL’s mission is to scientifically validate the traditional uses of medicinal plants for primary health care [28]. The interviews were conducted in the form of discussions in order to collect information about medicinal plants used for respiratory diseases, influenza, colds, and COVID-19. All participants provided oral prior informed consent before the interviews. The participants were asked to share their experiences (or those of relatives) of the plants they used for the mentioned ailments. During these interviews, the local names of the plants used in the treatment of the conditions, the parts of the plants, the route of administration, and the methods of preparation were recorded on a Microsoft Excel page. During the survey, photos of some plants were requested and provided by the interviewees over the phone. In the process, the plant specimens concerned were collected and then stored in the herbarium of the biomolecule department of COVACHIM-M2E, Faculty of Science, University of West Indies. The identity of each plant species mentioned by the families was verified and their scientific names confirmed by the books: plantes médicinale en Haïti [29], pharmacopée caribéenne [28] and flore illustrée des phanérogames de Guadeloupe et de Martinique 1 and 2 [100], the site Tramil [94] and the botanist at the University of West Indies.

The sampling technique used in this study is stratified sampling. In stratified sampling, the entire population U of N units is divided into a number (K) of mutually exclusive and exhaustive groups, which are called strata [131], each of which corresponds to a commune. The sample size (N = 120) has been selected as being representative of the population. From each of the strata, samples of suitable sizes are selected independently by some suitable sampling design. Eligible participants were people ages ≥ 18 years, Haitian living in the country for 18 years before. People were excluded if they had a disability that prevented them from hearing, understanding, or answering questions, and people who were members of the same family.

### 4.3. Data Analysis

Relative frequency of citation (RFC) was used to demonstrate the importance of each species. RFC was calculated with this formula: RFC = FC/N; where FC is the number of families mentioning the use of the species, and N is the total number of families participating in the survey [132].

The use value (UV) demonstrates the relative importance of the suggested plant species. UV was calculated using the following formula: UV = (∑Ui)/N where Ui is the number of use statements cited for a given plant species and N is the total number of families surveyed [132]. The UV was useful in determining the most commonly used plant species; these were those that were most frequently indicated in the treatment of a condition [132].

The family use value (FUV) was calculated to identify the importance of medicinal plant families. It was calculated as FUV = ∑UVs/ns × 100 where UVs: use values of the species, and ns: total number of species within each family [133].

The Informant Consensus Factor (ICF) was calculated to test for homogeneity of information using the following formula [134]: ICF = (Nur − Nt)/(Nur − 1) With Nur is the number of utilization reports for each disease category and Nt is the number of plant species used. The diseases were classified as respiratory, influenza, cold, and COVID-19 in order to calculate the ICF. The ICF is the value suggestive of how uniformly participants agree on the use of a particular plant species to treat a particular disease category. The ICF value ranges from 0 to 1. A value near 1 indicates that few plant species were used by a large group of participants. Alternatively, a low value indicates disagreement among participants regarding the use of a plant species for the treatment of a specific disease within a disease category. Medicinal plants presumed to be effective in the treatment of a certain disease have higher ICF values.

The level of fidelity (FL) of each plant was first determined, i.e., the ratio of the number of families that independently suggested the use of a species for the same diseases (Ip) to the total number of families that mentioned the plant for a major disease (Iu). FL was calculated by the following formula [135]: FL = (Ip × 100)/Iu. A high FL value means that the plant species was frequently used to treat a specific disease category by the participants.

## 5. Conclusions

According to the TRAMIL methodology, medicinal plant uses that are mentioned with a frequency of 20% or more are considered significant. Our study, conducted in Haiti, revealed that several plants are commonly used to treat influenza, colds, and respiratory diseases and are also used for COVID-19. Most of the frequently cited plants were tested for their antibacterial and/or antiviral activities, but the molecules responsible for these activities have not been identified or isolated for most of these plants. Additionally, *Samyda dodecandra* rosea Sims, which is the most commonly cited plant for influenza treatment in Haiti, has not been tested for biological activity in the literature. Therefore, it is recommended that further pharmacological studies be conducted on these plants.

We want to highlight that in this study, the intellectual property of indigenous knowledge owners and participants is explicitly recognized in accordance with the Nagoya Protocol on Access and Benefit Sharing of the Convention on Biological Diversity.

## Figures and Tables

**Table 1:** Sociodemographic factors related to the respondents.

		Male	% of Male	Female	% of Female
Gender		22	18.33	98	81.67%
Age (mean ± sd) years				39.29 ± 14.07	
Communes	Zone	Number of family representatives	% of family reprensentatives	Age	
Carrefour	Urban	5	4.17	24–67	
Croix-des-Bouquet	Rural	10	8.33	18–56	
Delmas	Urban	43	35.83	28–51	
Gressier	Rural	6	5	45–60	
Léogâne	Rural	6	5	21–70	
Pétion-Ville	Urban	20	16.67	23–70	
Port-au-Prince	Urban	20	16.67	19–69	
Tabarre	Rural	10	8.33	25–56	

**Table 2:** Percentage (%) of botanical families most used for all these preventions and remedies.

Botanical Families	% Respiratory Diseases	% Flu and Cold	% COVID-19
Amaryllidaceae	0.88	25.66	73.45
Asteraceae	5.5	49.54	44.95
Curcubitaceae	-	7.61	92.39
Lamiaceae	9.39	57.14	33.47
Myrtaceae	25	21	54
Rutaceae	8.13	51.20	40.67
Zingiberaceae	10.68	11.65	77.67

**Table 3:** The list of plants mentioned, Creole vernacular names, families, their RFC value, and their traditional uses. RFC = relative frequency of citation. N/A = not available. Traditional plant for flu, COVID-19, and respiratory disease. The voucher specimens are stored at the COVACHIM laboratory in Guadeloupe and, for some, at the URE laboratory at the Faculty of Sciences (UEH) in Haiti.

Scientific Names	Creole Vernacular Names	English Common Names	Families	Vouchers Specimen Codes	Number of Families Citing It in the Survey	RFC	Parts Used and Mode of Preparation Flu, Cold, Respiratory Diseases and for a Few COVID-19 Symptoms	References
*Allium ampeloprasum* var. porrum.	Pwawo	Leek	Liliaceae	URE91	2	0.016	Is used in infusions and/or decoction mixtures for COVID-19	N/A
*Abrus precatorius* L.	Lyann reglis	Rosary pea	Fabaceae	URE73	27	0.225	Decoction of leaves	N/A
*Allium cepa* L.	Zonyon	Onion	Amaryllidaceae	URE111	61	0.508	Mix the onion and take the juice with honey/or brown sugar for flu, cold	[28]
*Allium sativum* L.	Lay	Garlic	Amaryllidaceae	URE68	52	0.433	The garlic syrup to use against the or the juice with honey for flu, or bulb in decoction for flu or the stomach pains	[3,28]
*Aloe vera* L.	Lalwa	Aloe	Aloeaceae	URE63	63	0.525	Swallow a small butcher of aloe gel with water all morning for COVID-19 or gel in decoction for cold	[28,29]
*Ananas comosus* L.	Anana	Pineapple	Bromeliaceae	URE05	1	0.008	Fruit juice	N/A
*Annona muricata* L.	Kowosol	Soursop	Annonaceae	URE61	28	0.233	Leaf infusion/fruit juice	N/A
*Apium graveolens* L.	Seleri	Celery	Apiaceae	URE95	8	0.067	For flu syndrome, used fresh leafy stems in infusions and/or decoction	[28]
*Argemone mexicana* L.	Chadwon	Prickly Poppy	Papaveraceae	URE24	25	0.208	The root is used for stomach pains and 3 or 4 leaves and put them in a decoction for flu	[28,29]
*Artemisia vulgaris* L.	Amwaz	Mugwort	Asteraceae	URE04	47	0.392	Leaves infusion for COVID-19	N/A
*Bromelia pinguin* L.	Grenn pengwen	Pinuela	Bromeliaceae	URE39	1	0.008	Leaf decoction or grilled fruit	N/A
*Carnavalia rosea* L.	Lyann kayiman	Coastal Jack Bean	Fabaceae	URE71	8	0.067	Leaves and stems infusion/decoction for COVID-19	N/A
*Catalpa longissima* L.	Bwad chèn	Spanish Oak	Bignoniaceae	URE22	1	0.008	Bark in decoction for stomach pain and fever	[28]
*Cinnamomum verum* J. S. Presl	Kanèl	Cinnamon	Lauraceae	URE51	49	0.408	Bark decoction for vomiting and diarrhea	[28,30]
*Cissus sicyoides* L.	Lyann mòl	Princess vine	Vitaceae	URE72	23	0.192	Leaves in decoction	[28]
*Citrus aurantiifolia* (Christm.) Swingle	Sitwon	Lime	Rutaceae	URE98	59	0.492	Bark or leaf in decoction or infusion for fever. Leaf in decoction for headaches. Juice for flu, cough, and diarrhea	[31]
*Citrus aurantium* L.	Zoranj si	Bitter Orange	Rutaceae	URE84	47	0.392	Bark or leaf in decoction or infusion for fever. Leaf in decoction for flu. Juice for flu, cough, and diarrhea	[3,28,32]
*Citrus maxima* Merr.	Chadèk	Pomelo	Rutaceae	URE23	37	0.308	Leaves in decoction for fever	[3,28]
*Citrus reticulata* Blanco	Mandarin	Mandarin	Rutaceae	URE75	28	0.233	Fruit juice	N/A
*Citrus sinensis* L.	Zoranj dous	Sweet Orange	Rutaceae	URE112	38	0.317	Fruit juice and/or leaf in decoction for flu or cough. The bark in decoction for fever	[28,29]
*Cocos nucifera* L.	Kokoye	Coconut	Arecaceae	URE58	2	0.017	For flu, the oil of the fruit is used in friction on the chest. Also used against asthma by mouth	[28]
*Corchorus siliquosus* L.	Ti lalo	Slippery Dick	Malvaceae	URE102	12	0.1	Leaves and stems infusion/decoction	[29]
*Croton flavens* L.	Ti bonm	Welensali	Euphorbiaceae	URE101	1	0.008	Leaves in infusion	N/A
*Curcuma longa* L.	Safran	Turmeric	Zingiberaceae	URE94	8	0.067	Is used in infusions and/or decoction mixtures for COVID-19 and is also used in punch	N/A
*Cymbopogon citratus* (DC) Stapf	Sitwonèl	Lemongrass	Poaceae	COVA21	38	0.317	Leaves and/or root in decoction for flu, cold, and fever	[28]
*Daucus carota* subsp. Sativus	Kawót	Carrot	Apioideae	URE54	8	0.067	The juice	N/A
*Eucalyptus globulus* Labill.	Ekaliptis	Bluegum eucalyptus	Myrtaceae	URE29	39	0.325	Infusion and/or decoction of the leaves for flu, cold/leaves for internal infusion and inhalation	[28]
*Eupatorium odoratum* L.	Langichat	Siam weed	Asteraceae	URE65	50	0.417	Juice of the leaves to drink in a spoon/infusion of the leaves	[3,29]
*Grossypium barbadense* L.	Koton	Creole cotton	Malvaceae	URE59	1	0.008	Aerial parts or leaves in decoction for abdominal pains	[28]
*Guazuma ulmifolia* Lam.	Bwa dòm	Bastard Cedar	Malvaceae	URE20	12	0.1	Leaves in decoction for flu and cold	[28]
*Hamelia patens* Jacq.	Fèy koray	Scarlet bush	Rubiaceae	URE35	4	0.033	Natural leaf applied on the head or in bath	[28]
*Hibiscus rosa-sinensis* L.	Choublak	China-rose	Malvaceae	URE25	33	0.275	For flu and cold: infusion and/or decoction of young leaves/infusion of flowers for children. Crumpled leaf applied locally for headaches	[28,29]
*Hyptis suaveolens* L. Poit.	Gwo ten	Wild spikenard	Lamiaceae	URE41	32	0.267	Leaves in infusion or decoction	[29]
*Jatropha gossypifolia* L.	Ti medsiyen	Bellyache bush	Euphorbiaceae	URE103	25	0.208	Leaves in infusion or decoction for diarrhea and pain	[28]
*Jatropha curcas* L.	Gwo medsiyen	Physic Nut	Euphorbiaceae	URE79	1	0.008	Fresh leaf for bath	[28]
*Kalanchoe pinnata* (Lam,) Pers.	Fèy chòche	Cathedral bells	Crassulaceae	URE30	2	0.017	Leaves in decoction or juice for flu or cold. For headaches, the leaves are crushed and applied to the forehead	[28]
*Lantana camara* L.	Bonbonyen	Lantana	Verbenaceae	COVA14	13	0.108	Infusion and/or decoction of the leaves, flowers, or the aerial part for flu and cold	[28]
*Lipia alba* L.	Melis	Bushy matgrass	Verbenaceae	COVA08	39	0.325	Fresh leaves or aerial parts in decoction for flu, cold, and tiredness	[28]
*Lycopersicon Esculentum* Mill.	Tomat	Garden tomato	Solanaceae	URE105	24	0.2	Buds in infusion	[29]
*Malpighia emarginata* DC	Seriz	Acerola cherry	Malpighiaceae	URE96	38	0.317	Leaves in decoction/fruit juice	[29]
*Mammea americana* L.	Abriko	Mammee apple	Clusiaceae	URE02	18	0.15	Fruit juice	N/A
*Melia azedarach* L.	Fèy lila	Chinaberry tree	Meliaceae	URE32	8	0.067	Infusion of young leaves	[29]
*Melicoccus bijugatus* Jacq.	Kenèp	Mamoncillo	Sapindaceae	URE55	16	0.133	Gargle leaf juice/fruit food	[29]
*Mentha arvensis* L.	Fèy mant	Corn Mint	Lamiaceae	URE33	26	0.217	Infusion and/or decoction of the leaves for flu, cold, and stomach pains	[28]
*Mentha nemorosa* Wild.	Mant	Mentha nemorosa	Lamiaceae	URE76	38	0.317	Leafy stem in hot infusion	[29]
*Moringa oleifera* L.	Doliv	Horseradish tree	Moringaceae	URE17	25	0.208	Flower in hot infusion	N/A
*Mormordica charantia* L.	Asowosi/asosi	Bitter Gourd	Curcubitaceae	COVA12	92	0.767	The aerial parts crushed and/or aqueous maceration, in baths, frictions, and local application. Or the aerial parts are used in decoction for fever and pain	[3,28]
*Musa x paradisiaca* L.	Bannann	Plantain	Musaceae	URE12	24	0.2	Leaf in decoction/fruit food	[29]
*Myristica fragrans* L.	Miskad	Nutmeg	Myristicaceae	COVA04	13	0.108	Is used in infusions and/or decoction mixtures for COVID-19 and is also used in punch	N/A
*Ocimum basilicum* L.	Bazilik	Basil	Lamiaceae	URE15	39	0.325	Leaves in decoction for stomach pains and vomiting	[28]
*Ocimum gratissimum* L.	Fonbazen	Clove basil	Lamiaceae	URE09	7	0.058	Leaf in decoction for pain	[3,28]
*Ocimum micranthum* Wild.	Atiyayo	N/A	Lamiaceae	URE110	29	0.242	Leaf juice	N/A
*Opuntia antillana* Britton & Rose	Rakèt	Prickly pear	Cactaceae	URE93	1	0.008	3 small pieces to put in 1 L of water and drink it all day	N/A
*Passiflora edulis* Sims	Genadya	Passion fruit	Passiforaceae	URE37	30	0.25	Fruit juice	N/A
*Passiflora foetida* L.	Marigouya	Wild maracuja	Passiforaceae	URE77	12	0.1	Leaves in decoction	[29]
*Petroselinum sativum* Hoffm.	Pèsi	Parsley	Apiaceae	URE88	5	0.042	Is used in infusions and/or decoction mixtures for COVID-19	N/A
*Phaseolus vulgaris* L.	Pwa	French bean	Fabaceae	URE90	34	0.283	Leaves in decoction	[29]
*Phyllanthus niruri* L.	Dèyèdo	Gale Of Wind	Phyllanthaceae	URE28	40	0.333	Infusions and/or decoction for the fever	[29]
*Physalis angulata* L.	Kòkmòl	Balloon cherry	Solanaceae	URE57	6	0.05	Infusion of young leaves	[29]
*Pinus occidentalis* Sw.	Bwa pen/bwa chandèl	N/A	Pinaceae	URE21	12	0.1	Resin in infusion or essence in friction	[29]
*Plectranthus amboinicus* (Lour.) Spreng.	Gwo ten	Indian borage	Lamiaceae	COVA07	39	0.325	Infused leaves for cold	[28]
*Portulaca oleracea* L.	Koupye	Purslane	Portulacaceae	URE60	20	0.167	The leaves are edible	N/A
*Prosopis juliflora* Sw DC	Bayawond	Mesquite	Mimosaceae	URE14	24	0.2	Juice of leaves	[29]
*Rhoeo spathacea* (Sw.) Stearn.	Boul di mars	Boat lily	Commelinaceae	URE19	24	0.2	Leaves in infusion	[29]
*Ricinus communis* L.	Fèy maskreti	Castor bean	Euphorbiaceae	COVA18	39	0.325	Oil for fiction	[3,28,29]
*Saccharum officinarum* L.	Kann	Sugar cane	Poaceae	URE50	12	0.1	Syrup/stem juice	N/A
*Samyda rosea* Sims	Kasesèk/magrip	Guayabilla	Salicaceae	URE52	61	0.508	Infusion and/or decoction of the leaves	[29]
*Senecioides cinerea* L. O. Ktze	Tòchon sal	N/A	Asteraceae	URE104	12	0.1	Aerial part in decoction	[29]
*Stachytarpheta jamaicensis* (L.) Vahl.	Vèvenn	Blue porter weed	Verbenaceae	URE108	17	0.142	Is used in infusions and/or decoction for headaches	[3]
*Stemodia durantifolia* L.	Twa zòm fò	Whitewoolly twintip	Plantaginaceae	URE106	7	0.058	Aerial parts in decoction	[29]
*Syzygium aromaticum* L.	Jiwòf	Clove	Myrtaceae	URE44	61	0.508	Nail decoction for inhalation and/or orally	N/A
*Thymus vulgaris* L.	Ten	Thyme	Lamiaceae	URE99	25	0.208	Plant in decoction and/or infusion	[29]
*Verbascum thapsus* L.	Molenn	Big taper	Scrophulariaceae	URE81	12	0.1	Leaves and flowers in infusion	[29]
*Waltheria indica* L.	Bale blan	Sleepy morning	Malvaceae	URE10	12	0.1	Leaves in infusion or decoction	[29]
*Zingiber officinale* Roscoe	Jenjanm	Ginger	Zingiberaceae	URE43	95	0.792	Decoction, boil the root of the grated ginger for flu, cold, and fever	[28,29]

**Table 4:** Some oral herbal preparations for the prevention and treatment of COVID-19. Some remedies used to prevent/treat COVID-19 by Haitians using up to 6 plants in mixtures or punch with other ingredients.

Scientific Names	Amount of Plants Used	Mode of Preparation	References
*Allium ampeloprasum* var. porrum. & *Apium graveolens* L. & *Daucus carota* subsp. Sativus	3	One leek, one handful of celery, 2 carrots. Mix all together and drink the juice	N/A
*Allium sativum* L. & *Mormordica charantia* L. & *Phyllanthus niruri* L.	3	Decoction of a few cloves of garlic with the skin, then add the leaves of the Bitter Gourd and Gale Of Wind	[33]
*Allium cepa* L. & *Allium sativum* L. & *Aloe vera* L. Burn & *Myristica fragrans* & *Petroselinum sativum Hoffm.* & *Zingiber officinale* Roscoe	6	Mix a spoonful of aloe gel with a few cloves of garlic, nutmeg, parsley and ginger	[33]
*Allium cepa* L. & *Allium sativum* L & *Aloe vera* L. Burn & *Syzygium aromaticum* & *Zingiber officinale* Roscoe	5	Mix half a purple onion, 3 cloves of garlic, a spoonful of aloe gel, a pinch of powdered clove, and a piece of ginger to drink a spoonful twice a day	[33]
*Allium cepa* L. & *Aloe vera* L. Burn & *Myristica fragrans*	3	punch with milk and an egg, a large spoonful of aloe gel	[33]
*Allium sativum* L & *Aloe vera* L. Burn & *Cinnamomum verum* L. & *Zingiber officinale* Roscoe	4	Punch with milk, egg, and rum	[33]
*Allium sativum* L & *Cinnamomum verum* L. & *Citrus aurantiifolia* Swingle & *Mormordica charantia* L. & *Syzygium aromaticum* & *Zingiber officinale* Roscoe	6	Boil Bitter Gourd and chop a compartment of garlic, then add a few grains of clove and cinnamon, then prick a lime, and finally add a little ginger and put the whole in decoction. After boiling, before drinking it, squeeze the lemon juice	[33]
*Allium sativum* L & *Cinnamomum verum* L. & *Curcuma longa* L. & *Mormordica charantia* L. & *Syzygium aromaticum*	5	Decoction of 5 garlic cloves with a piece of turmeric and some peels of cinnamon, and then add the leaves of Bitter Gourd after a few minutes of boiling	[33]
*Allium sativum* L. & *Cinnamomum verum* L. & *Mormordica charantia* L. & *Eupatorium odoratum* L. & *Syzygium aromaticum* & *Zingiber officinale* Roscoe	6	Decoction of garlic pods with cinnamon bark and clove grains with ginger root, and after a few minutes of boiling, it is necessary to add the leaves of Bitter Gourd and siam weed	[33]
*Allium sativum* L. & *Citrus aurantiifolia* Swingle & *Zingiber officinale* Roscoe	3	Boil 2 to 3 cloves of garlic, the lime with its skin, and the grated ginger	[33]
*Allium sativum* L. & *Aloe vera* L. Burn	2	1 big spoon of aloe gel, then add half a glass of white rum and let macerate for 5 mn. Then, in a mixer, we put 5 lay pods and a spoon of honey syrup. Then, when the 5 mn approximately has elapsed we put the aloe to macerate with the rum in the mixer. Also, immediately after, we add half a glass of water proportioned to the rum and let mix the whole for 6 to 7 mn	[33]
*Aloe vera* L. Burn	1	Swallow a small butcher of aloe gel with water all morning	[33]
*Aloe vera* L. Burn & *Zingiber officinale* Roscoe	2	2 or 3 pieces of ginger plus 6 cloves of garlic plus a purple onion, we mix them, and we take only the juice after sieving, and we take one or two spoons	N/A
*Ananas comosus* L.	1	fruit juice/decoction fruit skin	N/A
*Artemisia vulgaris* L.	1	Boil 1 to 2 L of water and pour the hot water on a small amount of the plant leaf because it is very strong	[33]
*Carnavalia rosea* L.	1	Infusion of the stem with young leaves	N/A
*Catalpa longissima* L.	1	Infusion and/or decoction of the leaf	N/A
*Cinnamomum verum* L. & *Citrus aurantiifolia* Swingle & *Mormordica charantia* L. & *Phyllanthus niruri* L. & *Zingiber officinale* Roscoe	5	Boil some cinnamon peels, grated ginger and lime with skin for about 8 min, then add the leafy stems Bitter Gourd and the leafy stems Gale of wind	[33]
*Cinnamomum verum* L. & *Citrus aurantium* L. & *Syzygium aromaticum* L.	5	Boil some cinnamon bark, bitter orange without the skin and cloves	[33]
*Cinnamomum verum* L. & *Citrus aurantium* L. & *Mormordica charantia* L. & *Petroselinum sativum* Hoffm. & *Syzygium aromaticum* L. & *Zingiber officinale* Roscoe	6	Boil some cinnamon, grated ginger, bitter orange without the skin, and some cloves. Then add the parsley leaves and bitter gourd	[33]
*Cinnamomum verum* L. & *Plectranthus amboinicus* (Lour.) Spreng. & *Syzygium aromaticum* L. & *Zingiber offinale* Roscoe	4	Boil some cinnamon bark, grated ginger, and cloves. Then add the leaves of the Indian borage	[33]
*Cinnamomum verum* L. & *Syzygium aromaticum* L.	2	Decoction of clove seeds and cinnamon bark to drink every morning and evening	[33]
*Cinnamomum verum* L. & *Zingiber officinale* Roscoe	2	Mixed tea in decoction: Boil some cinnamon bark, grated ginger	[33]
*Cinnamomum verum* L.& *Croton flavens* L.	2	decoction of the cinnamon, then the leaf for about 2 min to drink twice a week for 1 month, then once a week	N/A
*Cinnamomum verum* L.& *Cymbopogon citratus* (DC) Stapf & *Zingiber officinale* Roscoe	3	Tea mixed in decoction. Boil for about 10 min the grated ginger, the cinnamon bark, and the leaf and/or the root of the lemongrass	[33]
*Citrus aurantiifolia* Swingle & *Mormordica charantia* L & *Phyllanthus niruri* L. & *Stachytarpheta jamaicensis* (L.) Vahl.	4	Tea mixed in decoction. Boil the lime, then add the leaves of Bitter Gourd, blue porter weed, and Gale Of Wind and let them boil for about 5 min	[33]
*Citrus aurantiifolia* Swingle & *Mormordica charantia* L.	2	Tea mixed in decoction. Boil the lime and then add the Bitter Gourd leaves	[33]
*Citrus aurantiifolia* Swingle & *Mormordica charantia* L. & *Syzygium aromaticum* L.	3	Tea mixed in decoction. Boil the lime and cloves, then add the Bitter Gourd leaves	[33]
*Citrus aurantiifolia* Swingle & *Mormordica charantia* L. & *Thymus vulgaris* L.	3	Tea mixed in decoction. Boil the lime and then add the thyme leaves and Bitter Gourd	[33]
*Citrus aurantiifolia* Swingle & *Phyllanthus niruri* L.	2	decoction of the whole plant with the fruit of the lemon with the skin	[33]
*Citrus aurantium* L. & *Cymbopogon citratus* (D.C) Stapf & *Syzygium aromaticum* L.	3	decoction of the bitter orange without the skin with lemongrass leaves and some cloves	[33]
*Cymbopogon citratus* (DC) Stapf	1	Decoction of the whole plant or leaf	N/A
*Eupatorium odoratum* L.	1	Infusion of the stem with leaf or a spoon of the juice of the leaf	N/A
*Melia azedarach/Azadirachta indica* A. Juss	1	Infusion of young leaves	N/A
*Mormordica charantia* L.	1	Infusion and/or decoction of the whole plant or maceration in rum	[33]
*Petroselinum sativum* Hoffm. & *Syzygium aromaticum* L. & *Zingiber officinale* Roscoe	3	Tea mixed in decoction. Boil the ginger and cloves, then add the parsley leaves	N/A
*Portulaca oleracea* L.	1	Eat as a vegetable by heating it just a little in water	N/A
*Samyda rosea* Sims	1	infusion/decoction of the leaf	N/A
*Stemodia durantifolia*	1	Infusion with a small quantity of the leaves because the plant is very strong	N/A
*Thymus vulgaris* L.	1	Infusion and/or decoction of the whole plant. Or put 2 tablespoons of powdered thyme, add hot water, let it wait for 4–5 min, then cut a lemon, squeeze it, and add 2 spoonfuls of honey	[33]
*Zingiber officinale* Roscoe	1	Decoction: Cut the ginger into small pieces or use a grater. Boil it for 10 to 15 min. When it is good, add honey or brown sugar	[33]

**Table 5:** List of herbal preparations and related information used for the treatment of COVID-19.

Plant Species	Number of Families Reporting the Use of Species	Number of Ailments Treated by the Species	Main Use	Number of Families for the Main Use	FL %
*Abrus precatorius* L.	27	1	Flu	27	100
*Allium cepa* L.	61	2	COVID-19	34	55.737
*Allium sativum* L.	52	3	COVID-19	49	94.231
*Aloe vera* L.	63	1	COVID-19	63	100
*Annona muricata* L.	28	3	Flu	16	57.143
*Argemone mexicana* L.	25	1	Flu	25	100
*Artemisia vulgaris* L.	47	1	COVID-19	47	100
*Cinnamomum verum* J. S. Presl	49	2	Flu	30	61.224
*Cissus sicyoides* L.	23	1	Flu	23	100
*Citrus aurantifolia* Swingle	59	3	Flu	31	52.542
*Citrus aurantium* L.	47	3	Flu	33	70.213
*Citrus maxima Merr.*	37	2	COVID-19	22	59.459
*Citrus reticulata Blanco*	28	2	COVID-19	18	64.286
*Citrus sinensis* L.	38	3	Flu	18	47.364
*Corchorus siliquosus* L.	12	1	Flu	12	100
*Cymbopogon citratus* (DC) Stapf.	38	2	Flu	27	71.053
*Eucalyptus globulus Labill.*	39	3	Flu	20	51.282
*Eupatorium odoratum* L.	50	3	Flu	42	84
*Guazuma ulmifolia* Lam.	12	1	Flu	12	100
*Hibiscus rosa-sinensis* L.	33	1	Flu	33	100
*Hyptis suaveolens* L. Poit.	32	2	COVID-19	17	53.125
*Jatropha gossypiifolia* L.	25	2	Flu	24	96
*Lantana camara* L.	13	1	Flu	13	100
*Lipia alba* L.	39	10	Flu	29	74.359
*Lycopersicon Esculentum* Mill.	24	2	Flu/COVID-19	12	50
*Malpighia emarginata* DC	38	2	Flu	35	92.105
*Mammea americana* L.	18	2	Flu	11	61.11
*Melicoccus bijugatus* Jacq	16	2	Flu	12	75
*Mentha arvensis* L.	26	3	COVID-19	11	42.307
*Mentha nemorosa* Wild.	38	2	Flu	34	89.473
*Moringa oleifera* L.	25	2	COVID-19	15	60
*Mormordica charantia* L.	92	2	COVID-19	85	92.391
*Musa x paradisiaca* L.	24	1	Flu	24	100
*Myristica fragrans* L.	13	2	Flu	8	61.538
*Ocimum basilicum* L.	39	3	Flu	25	64.102
*Ocimum micranthum* Wild.	29	2	Flu	24	82.758
*Passiflora edulis* Sims	30	2	Flu	21	70
*Passiflora foetida* L.	12	1	Flu	12	100
*Phaseolus vulgaris* L.	34	2	Flu	24	70.588
*Phyllanthus niruri* L.	40	1	COVID-19	40	100
*Pinus occidentalis* Sw.	12	1	Flu	12	100
*Plectranthus amboinicus* (Lour.) Spreng	39	2	Flu	27	69.231
*Portulaca oleracea* L.	20	1	COVID-19	20	100
*Prosopis juliflora* Sw. DC	24	1	Flu	24	100
*Rhoeo spathacea* (Sw.) Steam	24	1	Flu	24	100
*Ricinus communis* L.	39	3	Respiratory diseases	15	38.461
*Saccharum officinarum* L.	12	1	Flu	12	100
*Samyda dodecandra* Jacq.	61	3	Flu	59	96.721
*Senecioides cinerea* L. O. Ktze	12	1	Flu	12	100
*Stachytarpheta jamaicensis* (L.) Vahl.	17	1	COVID-19	17	100
*Syzygium aromaticum* L.	61	3	COVID-19	50	81.967
*Thymus vulgaris* L.	25	2	COVID-19	17	68
*Verbascum thapsus* L.	12	1	Flu	12	100
*Waltheria indica* L.	12	1	Flu	12	100
*Zingiber officinale* Roscoe	95	3	COVID-19	72	75.789

**Table 6:** The list of plants mentioned and parts used. N/A = not available.

Scientific Names	Families	Parts Used in the Literature	Uses in the Literature	References
*Allium ampeloprasum* var. porrum.	Liliaceae	Bulbs	Inflammatory, gastroprotective activities	[35]
*Abrus precatorius* L.	Fabaceae	Seeds, fresh roots	Antifungal, apoptotic, antihistaminic, antiproliferative, anti-oxidant, potential anticancer agents	[36]
*Allium cepa* L.	Amaryllidaceae	Bulbs	Anti-oxidant, immuno-modulator, antimicrobial, neuroprotective properties	[37]
*Allium sativum* L.	Amaryllidaceae	Bulbs	For colds, fever, cough, asthma, antimicrobial	[38]
*Aloe vera* L.	Aloeaceae	Leaves, gel	Antiviral, antidiabetic, laxative, protective against radiation, antiallergic, anti-inflammatory, antibacterial	[39]
*Ananas comosus* L.	Bromeliaceae	Peel, fruit, leaf	Antimalarial, anti-nociceptive and anti-inflammatory	[40]
*Annona muricata* L.	Annonaceae	Plants, leaves,	Anti-angiogenic, antibacterial, antiviral, anti-inflammatory, anti-oxidant	[41]
*Apium graveolens* L.	Apiaceae	Plant, root, leaf	Antimicrobial, antiparasitic, cardioprotective, gastroprotective, neuroprotective, hypolipidemic, cytotoxic, anti-oxidant, anti-inflammatory, and anti-infertility	[42]
*Argemone mexicana* L.	Papaveraceae	Flowers, berries, and leaves	Antibacterial and antifungal	[43]
*Artemisia vulgaris* L.	Asteraceae	Plant	Antimalarial, anti-inflammatory, antihypertensive, anti-oxidant, anti-tumoral, immunomodulatory, hepatoprotective, antispasmodic and antiseptic	[44]
*Bromelia pinguin* L.	Bromeliaceae	Fruit pulp	Antifungal	[45]
*Carnavalia rosea* L.	Fabaceae	Leaves	In Haiti, use against flu	N/A
*Catalpa longissimi* L.	Bignoniaceae	Leaf, plant, bark	In Haiti, use against pneumopathy, antidiarrheal, sore throats, stomach aches	[29]
*Cinnamomum verum* J. S. Presl	Lauraceae	Bark	Anti-oxidant, antimicrobial, anti-inflammatory	[30]
*Cissus sicyoides* L.	Vitaceae	Leaves and stems	Anti-oxidant activity, neuroprotective and anti-inflammatory	[46]
*Citrus aurantiifolia* (Christm.) Swingle	Rutaceae	Leaves and fruit peels	Antimicrobial, fungicidal, insecticidal, anticancer, anti-oxidant properties	[31]
*Citrus aurantium* L.	Rutaceae	Leaves, fruit, fruit peel, fruit peel essential oil	Anti-oxidant properties, antimicrobial, antifungal, antiparasitic, and anti-inflammatory activities	[32]
*Citrus maxima* Merr.	Rutaceae	Peel essential oil	Antibacterial	[47]
*Citrus reticulata* Blanco	Rutaceae	Peels	Anti-oxidant and antiproliferative	[48]
*Citrus sinensis* L.	Rutaceae	Seed oil, fruit	Anti-oxidant, antimicrobial	[49]
*Cocos nucifera* L.	Arecaceae	Floral axis, root, coconut water	Antibacterial, anti-oxidant	[50]
*Corchorus siliquosus* L.	Malvaceae	Leaves and stems	In Haiti, use against flu	[29]
*Croton flavens* L.	Euphorbiaceae	Leaf essential oil	Anticancer activity	[51]
*Curcuma longa* L.	Zingiberaceae	Curcumin of root	Antiviral, antitumor, anti-atherogenic, anti-inflammatory	[52]
*Cymbopogon citratus* (DC) Stapf	Poaceae	Leaves and roots	Antibacterial	[53]
*Daucus carota* subsp. Sativus	Apioideae	Aerial parts, oil	Antibacterial	[54]
*Eucalyptus globulus* Labill.	Myrtaceae	Essential oils, leaves	Antibacterial	[55]
*Eupatorium odoratum* L.	Asteraceae	Plant	antimicrobial, anti-inflammatory, analgesic, anti-oxidant, and cytoprotective properties	[56,57]
*Grossypium barbadense* L.	Malvaceae	Leaves and roots	Anti-oxidant, antidiarrheal, healing, diuretic, respiratory conditions, pneumonia	[58]
*Guazuma ulmifolia* Lam.	Malvaceae	Stem bark, fruit, and leaves	Antimicrobial, anti-oxidant, antiprotozoal, antidiarrheal activities, and cardioprotective effect	[59]
*Hamelia patens* Jacq.	Rubiaceae	Leaves	Antibacterial	[60]
*Hibiscus rosa-sinensis* L.	Malvaceae	Parts used and mode of preparation for flu, cold, and respiratory diseases	Antibacterial, anti-oxidant	[61]
*Hyptis suaveolens* L. Poit.	Lamiaceae	Essential oil of leaves, leaves	Antimicrobial, antiviral	[62]
*Jatropha gossypifolia* L.	Euphorbiaceae	Different parts	Antimicrobial	[63]
*Jatropha curcas* L.	Euphorbiaceae	Root, seed, stem, leaf, and flower	Antimicrobial	[64]
*Kalanchoe pinnata* (Lam,) Pers.	Crassulaceae	Leaves	Anti-inflammatory, antibacterial, anti-tumorous	[65]
*Lantana camara* L.	Verbenaceae	Leaves	Antibacterial	[66]
*Lipia alba* L.	Verbenaceae	Essential oils of leaf, aerial parts	Antibacterial	[67]
*Lycopersicon Esculentum* Mill.	Solanaceae	Tomatoside	Antiviral	[68]
*Malpighia emarginata* DC	Malpighiaceae	Fruit	Anti-oxidant activity and antimicrobial	[69]
*Mammea americana* L.	Clusiaceae	Fruit, leaves, fruit peel, seed	Antibacterial	[70]
*Melia azedarach* L.	Meliaceae	Leaves, root, and stem bark	Antibacterial	[71]
*Melicoccus bijugatus* Jacq.	Sapindaceae	Fruits	Antimicrobial	[72]
*Mentha arvensis* L.	Lamiaceae	Leaves	Antibacterial	[73]
*Mentha nemorosa* Wild.	Lamiaceae	Leafy stem	In Haiti, use against flu, anti-inflammatory	[29]
*Moringa oleifera* L.	Moringaceae	Leaves, roots, pods, seeds, and flowers	Antidiabetic, antibacterial, antifungal, and anti-carcinogenic	[74]
*Mormordica charantia* L.	Curcubitaceae	Leaves, seeds, whole plant	Antibacterial, anti-flu, anti-fever antidiabetic, anthelmintic, antiviral, anti-diarrhea	[11,12]
*Musa x paradisiaca* L.	Musaceae	Peel	Antibacterial	[75]
*Myristica fragrans* L.	Myristicaceae	Fruits	Antibacterial	[76]
*Ocimum basilicum* L.	Lamiaceae	Leaves essential oil	Antibacterial	[77]
*Ocimum gratissimum* L.	Lamiaceae	Leaves essential oil, clove	Antibacterial, anti-inflammatory, anticancer, hepatoprotective, antidiabetic, antihypertensive, antidiarrhoeal	[78]
*Ocimum micranthum* wild.	Lamiaceae	Essential oils	Antibacterial	[79]
*Opuntia antillana* Britton & Rose	Cactaceae	Leaf	Antibacterial, anti-oxidant	[80]
*Passiflora edulis* Sims	Passiforaceae	Fruits	Anxiolytic, anti-inflammatory, antibacterial	[81]
*Passiflora foetida* L.	Passiforaceae	Flower, leaf, fruit	Antibacterial, analgesic, and antidiarrhoeal	[82]
*Petroselinum sativum* Hoffm.	Apiaceae	Aerial part, plant	Anti-inflammatory, anti-oxidant, analgesic and spasmolytic, antidiabetic, immunomodulating, and gastrointestinal effects	[83]
*Phaseolus vulgaris* L.	Fabaceae	Seeds, homodimeric lectin from seed	Antibacterial, antifungal, and antiviral	[84]
*Phyllanthus niruri* L.	Phyllanthaceae	Leaves and fruits	Anti-fever, anti-diarrhea, anti-inflammatory	[85]
*Physalis angulata* L.	Solanaceae	Fruit juice, leaf	Antidiabetic, anti-inflammatory, antibacterial, anti-oxidant	[26]
*Pinus occidentalis* Sw.	Pinaceae	Resin	In Haiti, use against flu, antirheumatic	[29]
*Plectranthus amboinicus (Lour.) Spreng.*	Lamiaceae	Leaves	Anti-inflammatory, antitumor, antibacterial	[86]
*Portulaca oleracea* L.	Portulacaceae	Aerial parts	Antiviral, antibacterial, anti-inflammatory, antitumor	[8]
*Prosopis juliflora* Sw DC	Mimosaceae	Leaves, julifloravizole	Antifungal and antibacterial	[87]
*Rhoeo spathacea* (Sw.) Stearn.	Commelinaceae	Plant, leaves	Antifertility, anti-inflammatory, anti-oxidant, and antibacterial	[1]
*Ricinus communis* L.	Euphorbiaceae	Leaves, stems, roots, seeds and capsules	Antimicrobial, anti-oxidant, insecticidal, antiasthmatic, anti-inflammatory	[88]
*Saccharum officinarum* L.	Poaceae	Leaves	Antibacterial	[89]
*Samyda rosea* Sims	Salicaceae	Leaves	In Haiti used as an anti-flu/anti-cough	[29]
*Senecioides cinerea* L. O. Ktze	Asteraceae	Plant	In Haiti, use against flu/cold	[29]
*Stachytarpheta jamaicensis* (L.) Vahl.	Verbenaceae	Leaves	Antibacterial, antidiarrheal	[71]
*Stemodia durantifolia* L.	Plantaginaceae	Leaves	Antifertility	[1]
*Syzygium aromaticum* L.	Myrtaceae	Clove	Anti-oxidant and antimicrobial	[90]
*Thymus vulgaris* L.	Lamiaceae	Essential oils	Antibacterial	[91]
*Verbascum thapsus* L.	Scrophulariaceae	Leaves	Antibacterial, inflammatory diseases, diarrhea, asthma	[92]
*Waltheria indica* L.	Malvaceae		Anti-inflammatory, antiviral, anti-flu	[93]
*Zingiber officinale* Roscoe	Zingiberaceae	Root	Antimicrobial, anti-inflammatory, antiviral	[9,10]

## Data Availability

The original contributions presented in the study are included in the article, further inquiries can be directed to the corresponding authors.

## References

[1] Weniger B., Haag-Berrurier M., Anton R. (1982). Plants of Haiti used as antifertility agents. J. Ethnopharmacol..

[2] Danton O., Somboro A., Fofana B., Diallo D., Sidibé L., Rubat-Coudert C., Marchand F., Eschalier A., Ducki S., Chalard P. (2019). Ethnopharmacological survey of plants used in the traditional treatment of pain conditions in Mali. J. Herb. Med..

[3] Salla B. (2017). Prise en charge des symptômes douloureux par la médecine traditionnelle haïtienne: Résultats d’une enquête réalisée dans le quartier de Martissant à Port-au-Prince. Douleurs Éval. Diagn. Trait..

[4] Vital P.G., Rivera W.L. (2009). Antimicrobial activity and cytotoxicity of *Chromolaena odorata* (L. f.) King and Robinson and *Uncaria perrottetii* (A. Rich) Merr. Extracts. J. Med. Plants Res..

[5] Newman D.J., Cragg G.M. (2007). Natural Products as Sources of New Drugs over the Last 25 Years. J. Nat. Prod..

[6] Al-Sayed E., El-Naga R.N. (2015). Protective role of ellagitannins from *Eucalyptus citriodora* against ethanol-induced gastric ulcer in rats: Impact on oxidative stress, inflammation and calcitonin-gene related peptide. Phytomedicine.

[7] Gismondi A., Canuti L., Impei S., Di Marco G., Kenzo M., Colizzi V., Canini A. (2013). Antioxidant extracts of African medicinal plants induce cell cycle arrest and differentiation in B16F10 melanoma cells. Int. J. Oncol..

[8] Liu Y., Zhao L., Xie Y., Chen Z., Yang S., Yin B., Li G., Guo H., Lin S., Wu J. (2021). Antiviral activity of *Portulaca oleracea* L. extracts against porcine epidemic diarrhea virus by partial suppression on myd88/NF-κb activation in vitro. Microb. Pathog..

[9] Chang J.S., Wang K.C., Yeh C.F., Shieh D.E., Chiang L.C. (2013). Fresh ginger (*Zingiber officinale*) has anti-viral activity against human respiratory syncytial virus in human respiratory tract cell lines. J. Ethnopharmacol..

[10] Chrubasik S., Pittler M.H., Roufogalis B.D. (2005). *Zingiberis rhizoma*: A comprehensive review on the ginger effect and efficacy profiles. Phytomedicine.

[11] Pongthanapisith V., Ikuta K., Puthavathana P., Leelamanit W. (2013). Antiviral Protein of *Momordica charantia* L. Inhibits Different Subtypes of Influenza, A. Evid.-Based Complement. Altern. Med..

[12] Subratty A.H., Gurib-Fakim A., Mahomoodally F. (2005). Bitter melon: An exotic vegetable with medicinal values. Nutr. Food Sci..

[13] WHO (2019). WHO EMRO|Maladies Infectieuses|Thèmes de Santé. World Health Organization—Regional Office for the Eastern Mediterranean. http://www.emro.who.int/fr/health-topics/infectious-diseases/index.html.

[14] Lasky J.A., Fuloria J., Morrison M.E., Lanier R., Naderer O., Brundage T., Melemed A. (2021). Design and Rationale of a Randomized, Double-Blind, Placebo-Controlled, Phase 2/3 Study Evaluating Dociparstat in Acute Lung Injury Associated with Severe COVID-19. Adv. Ther..

[15] Wu X., Chen Q., Li J., Liu Z. (2021). Diagnostic techniques for COVID-19: A mini-review. J. Virol. Methods.

[16] WHO WHO Director-General’s Opening Remarks at the Media Briefing on COVID-19. 11 March 2020. https://www.who.int/director-general/speeches/detail/who-director-general-s-opening-remarks-at-the-media-briefing-on-covid-19---11-march-2020.

[17] La Rosa G., Bonadonna L., Lucentini L., Kenmoe S., Suffredini E. (2020). Coronavirus in water environments: Occurrence, persistence and concentration methods—A scoping review. Water Res..

[18] Setti L., Passarini F., De Gennaro G., Barbieri P., Perrone M.G., Borelli M., Palmisani J., Di Gilio A., Piscitelli P., Miani A. (2020). Airborne Transmission Route of COVID-19: Why 2 Meters/6 Feet of Inter-Personal Distance Could Not Be Enough. Int. J. Environ. Res. Public Health.

[19] Ong S.W.X., Tan Y.K., Chia P.Y., Lee T.H., Ng O.T., Wong M.S.Y., Marimuthu K. (2020). Air, Surface Environmental, and Personal Protective Equipment Contamination by Severe Acute Respiratory Syndrome Coronavirus 2 (SARS-CoV-2) from a Symptomatic Patient. JAMA.

[20] Meselson M. (2020). Droplets and Aerosols in the Transmission of SARS-CoV-2. N. Engl. J. Med..

[21] Qian H., Miao T., Liu L., Zheng X., Luo D., Li Y. (2021). Indoor transmission of SARS-CoV-2. Indoor Air.

[22] Jotz G.P., Voegels R.L., Bento R.F. (2020). Otorhinolaryngologists and Coronavirus Disease 2019 (COVID-19). Int. Arch. Otorhinolaryngol..

[23] Kosugi E.M., Lavinsky J., Romano F.R., Fornazieri M.A., Luz-Matsumoto G.R., Lessa M.M., Piltcher O.B., Sant’Anna G.D. (2020). Incomplete and late recovery of sudden olfactory dysfunction in COVID-19. Braz. J. Otorhinolaryngol..

[24] Pan L., Mu M., Yang P., Sun Y., Wang R., Yan J., Li P., Hu B., Wang J., Hu C. (2020). Clinical Characteristics of COVID-19 Patients With Digestive Symptoms in Hubei, China: A Descriptive, Cross-Sectional, Multicenter Study. Am. J. Gastroenterol..

[25] Sultana M.S., Khan A.H., Hossain S., Hasan M.T. (2021). Mental health difficulties in students with suspected COVID-19 symptoms and students without suspected COVID-19 symptoms: A cross-sectional comparative study during the COVID-19 pandemic. Child. Youth Serv. Rev..

[26] Nasir A., Shah M.A., Ashraf U., Khan A., Jeon G. (2021). An intelligent framework to predict socioeconomic impacts of COVID-19 and public sentiments. Comput. Electr. Eng..

[27] Petrakou K., Iatrou G., Lamari F.N. (2020). Ethnopharmacological survey of medicinal plants traded in herbal markets in the Peloponnisos, Greece. J. Herb. Med..

[28] TRAMIL (2014). Pharmacopée Végétale Caribéenne: Troisième Édition Actualisée Et Enrichie. TRAMIL. CANOPE de Guadeloupe, 2014. https://www.tramil.net/fr/content/publications-tramil.

[29] Neptune-Rouzier M. (2014). Plantes Médicinales d’Haïti: Description, Usages et Propriétés.

[30] Singh N., Rao A.S., Nandal A., Kumar S., Yadav S.S., Ganaie S.A., Narasimhan B. (2021). Phytochemical and pharmacological review of *Cinnamomum verum* J. Presl-a versatile spice used in food and nutrition. Food Chem..

[31] Weimer P., Moura J.G.L., Mossmann V., Immig M.L., de Castilhos J., Rossi R.C. (2021). *Citrus aurantiifolia* (Christm) Swingle: Biological potential and safety profile of essential oils from leaves and fruit peels. Food Biosci..

[32] Nidhi P., Rolta R., Kumar V., Dev K., Sourirajan A. (2020). Synergistic potential of Citrus aurantium L. essential oil with antibiotics against *Candida albicans*. J. Ethnopharmacol..

[33] Bien-Aimé A., Casimir J., Civil M.-F., Noël E., Rouzier M. Les Recettes Haïtiennes Face à la Pandémie Provoquée par le SARS-CoV-2 Par la Commission D’inventaire et D’évaluation des Remèdes Traditionnels Contre le SARS-CoV-2-UEH, Juillet 2020. https://ueh.edu.ht/wp-content/uploads/2020/08/Rapport-du-Jury-mis-sur-pied-par-lUEH-sur-les-recettes-traditionnelles-haitiennes-face-%C3%A0-la-Covid-19-1.pdf.

[34] Calcuttawala F. (2022). Nutrition as a key to boost immunity against COVID-19. Clin. Nutr. ESPEN.

[35] Adão C.R., da Silva B.P., Parente J.P. (2011). A new steroidal saponin from *Allium ampeloprasum* var. porrum with antiinflammatory and gastroprotective effects. Phytochem. Lett..

[36] Okoro E.E., Maharjan R., Jabeen A., Ahmad M.S., Azhar M., Shehla N., Zaman W., Shams S., Osoniyi O.R., Onajobi F.D. (2021). Isoflavanquinones from *Abrus precatorius* roots with their antiproliferative and anti-inflammatory effects. Phytochemistry.

[37] Kumar M., Barbhai M.D., Hasan M., Punia S., Dhumal S., Radha, Rais N., Chandran D., Pandiselvam R., Kothakota A. (2022). Onion (*Allium cepa* L.) peels: A review on bioactive compounds and biomedical activities. Biomed. Pharmacother..

[38] Torpol K., Wiriyacharee P., Sriwattana S., Sangsuwan J., Prinyawiwatkul W. (2018). Antimicrobia activity of garlic (*Allium sativum* L.) and holy basil (*Ocimum sanctum* L.) essential oils applied by liquid vs. vapour phases. Int. J. Food Sci. Technol..

[39] Farid A., Tawfik A., Elsioufy B., Safwat G. (2021). In vitro and in vivo anti-Cryptosporidium and anti-inflammatory effects of Aloe vera gel in dexamethasone immunosuppressed mice. Int. J. Parasitol. Drugs Drug Resist..

[40] Ajayi A.M., Coker A.I., Oyebanjo O.T., Adebanjo I.M., Ademowo O.G. (2022). *Ananas comosus* (L) Merrill (pineapple) fruit peel extract demonstrates antimalarial, anti-nociceptive and anti-inflammatory activities in experimental models. J. Ethnopharmacol..

[41] Balderrama-Carmona A.P., Silva-Beltrán N.P., Gálvez-Ruiz J.-C., Ruíz-Cruz S., Chaidez-Quiroz C., Morán-Palacio E.F. (2020). Antiviral, Antioxidant, and Antihemolytic Effect of *Annona muricata* L. Leaves Extracts. Plants.

[42] Khairullah A.R. (2021). Review on the Pharmacological and Health Aspects of Apium Graveolens or Celery: An Update. Syst. Rev. Pharm..

[43] Andleeb S., Alsalme A., Al-Zaqri N., Warad I., Alkahtani J., Bukhari S.M. (2020). In-vitro antibacterial and antifungal properties of the organic solvent extract of *Argemone mexicana* L.. J. King Saud Univ. Sci..

[44] Abiri R., Silva A.L.M., de Mesquita L.S.S., de Mesquita J.W.C., Atabaki N., de Almeida E.B., Shaharuddin N.A., Malik S. (2018). Towards a better understanding of *Artemisia vulgaris*: Botany, phytochemistry, pharmacological and biotechnological potential. Food Res. Int..

[45] Camacho-Hernández I.L., Chávez-Velázquez J.A., Uribe-Beltrán M.J., Rı A., Delgado-Vargas F. (2002). Antifungal activity of fruit pulp extract from *Bromelia pinguin*. Fitoterapia.

[46] Salazar M.A.R., Costa J.V., Urbina G.R.O., Cunha V.M.B., Silva M.P., do Nascimento Bezerra P., Pinheiro W.B.S., Gomes-Leal W., Lopes A.S., Carvalho Junior R.N. (2018). Chemical composition, antioxidant activity, neuroprotective and anti-inflammatory effects of cipó-pucá (*Cissus sicyoides* L.) extracts obtained from supercritical extraction. J. Supercrit. Fluids.

[47] Chen Y., Li T., Bai J., Nong L., Ning Z., Hu Z., Xu A., Xu C.-P. (2018). Chemical Composition and Antibacterial Activity of the Essential Oil of *Citrus maxima* (Burm.) Merr. cv. Shatian Yu. J. Biol. Act. Prod. Nat..

[48] Ferreira S.S., Silva A.M., Nunes F.M. (2018). *Citrus reticulata* Blanco peels as a source of antioxidant and anti-proliferative phenolic compounds. Ind. Crops Prod..

[49] Atolani O., Adamu N., Oguntoye O.S., Zubair M.F., Fabiyi O.A., Oyegoke R.A., Adeyemi O.S., Areh E.T., Tarigha D.E., Kambizi L. (2020). Chemical characterization, antioxidant, cytotoxicity, Anti-*Toxoplasma gondii* and antimicrobial potentials of the *Citrus sinensis* seed oil for sustainable cosmeceutical production. Heliyon.

[50] Shen X., Chen W., Zheng Y., Lei X., Tang M., Wang H., Song F. (2017). Chemical composition, antibacterial and antioxidant activities of hydrosols from different parts of *Areca catechu* L. and *Cocos nucifera* L.. Ind. Crops Prod..

[51] Sylvestre M., Pichette A., Longtin A., Nagau F., Legault J. (2006). Essential oil analysis and anticancer activity of leaf essential oil of *Croton flavens* L. from Guadeloupe. J. Ethnopharmacol..

[52] Kim H.J., Yoo H.S., Kim J.C., Park C.S., Choi M.S., Kim M., Choi H., Min J.S., Kim Y.S., Yoon S.W. (2009). Antiviral effect of *Curcuma longa* Linn extract against hepatitis B virus replication. J. Ethnopharmacol..

[53] Subramaniam G., Yew X.Y., Sivasamugham L.A. (2020). Antibacterial activity of *Cymbopogon citratus* against clinically important bacteria. S. Afr. J. Chem. Eng..

[54] Snene A., Mokni R.E., Mahdhi A., Joshi R.K., Hammami S. (2020). Comparative study of essential oils composition and in vitro antibacterial effects of two subspecies of *Daucus carota* growing in Tunisia. S. Afr. J. Bot..

[55] Bachir R.G., Benali M. (2012). Antibacterial activity of the essential oils from the leaves of *Eucalyptus globulus* against *Escherichia coli* and *Staphylococcus aureus*. Asian Pac. J. Trop. Biomed..

[56] Cui S., Tan S., Ouyang G., Jiang S., Pawliszyn J. (2009). Headspace solid-phase microextraction gas chromatography–mass spectrometry analysis of *Eupatorium odoratum* extract as an oviposition repellent. J. Chromatogr. B.

[57] Vijayaraghavan K., Rajkumar J., Seyed M.A. (2018). Phytochemical screening, free radical scavenging and antimicrobial potential of *Chromolaena odorata* leaf extracts against pathogenic bacterium in wound infections—A multispectrum perspective. Biocatal. Agric. Biotechnol..

[58] de Lima L.F., de Oliveira J.O., Carneiro J.N.P., Lima C.N.F., Coutinho H.D.M., Morais-Braga M.F.B. (2021). Ethnobotanical and antimicrobial activities of the Gossypium (Cotton) genus: A review. J. Ethnopharmacol..

[59] Pereira G.A., Araujo N.M.P., Arruda H.S., de P. Farias D., Molina G., Pastore G.M. (2019). Phytochemicals and biological activities of mutamba (*Guazuma ulmifolia* Lam.): A review. Food Res. Int..

[60] Paz J.E.W., Contreras C.R., Munguía A.R., Aguilar C.N., Inungaray M.L.C. (2018). Phenolic content and antibacterial activity of extracts of *Hamelia patens* obtained by different extraction methods. Braz. J. Microbiol..

[61] Mak Y.W., Chuah L.O., Ahmad R., Bhat R. (2013). Antioxidant and antibacterial activities of hibiscus (*Hibiscus rosa-sinensis* L.) and Cassia (*Senna bicapsularis* L.) flower extracts. J. King Saud Univ. Sci..

[62] Asekun O.T., Ekundayo O., Adeniyi B.A. (1999). Antimicrobial activity of the essential oil of *Hyptis suaveolens* leaves. Fitoterapia.

[63] Kumar V.P., Chauhan N.S., Padh H., Rajani M. (2006). Search for antibacterial and antifungal agents from selected Indian medicinal plants. J. Ethnopharmacol..

[64] Rahu M.I., Naqvi S.H.A., Memon N.H., Idrees M., Kandhro F., Pathan N.L., Sarker M.N.I., Bhutto M.A. (2021). Determination of antimicrobial and phytochemical compounds of *Jatropha curcas* plant. Saudi J. Biol. Sci..

[65] Agarwal H., Shanmugam V.K. (2019). Anti-inflammatory activity screening of *Kalanchoe pinnata* methanol extract and its validation using a computational simulation approach. Inform. Med. Unlocked.

[66] Dubey D., Padhy R.N. (2013). Antibacterial activity of *Lantana camara* L. against multidrug resistant pathogens from ICU patients of a teaching hospital. J. Herb. Med..

[67] da Silva Júnior A.Q., da Silva D.S., Figueiredo P.L.B., Sarrazin S.L.F., Bouillet L.E.M., de Oliveira R.B., Maia J.G.S., Mourão R.H.V. (2019). Seasonal and circadian evaluation of a citral-chemotype from *Lippia alba* essential oil displaying antibacterial activity. Biochem. Syst. Ecol..

[68] Spinu K., Waller G.R., Yamasaki K. (1996). Antiviral Activity of Tomatoside from *Lycopersicon esculentum* Mill. Saponins Used in Traditional and Modern Medicine.

[69] Delva L., Goodrich-Schneider R. (2013). Antioxidant activity and antimicrobial properties of phenolic extracts from acerola (*Malpighia emarginata* DC) fruit. Int. J. Food Sci. Technol..

[70] Lemus C., Smith-Ravin J., Marcelin O. (2021). *Mammea americana*: A review of traditional uses, phytochemistry and biological activities. J. Herb. Med..

[71] Khan M.M., Harunsani M.H., Tan A.L., Hojamberdiev M., Poi Y.A., Ahmad N. (2020). Antibacterial Studies of ZnO and Cu-Doped ZnO Nanoparticles Synthesized Using Aqueous Leaf Extract of *Stachytarpheta jamaicensis*. BioNanoScience.

[72] Bystrom L.M., Lewis B.A., Brown D.L., Rodriguez E., Obendorf R.L. (2009). Phenolics, Sugars, Antimicrobial and Free-Radical-Scavenging Activities of *Melicoccus bijugatus* Jacq. Fruits from the Dominican Republic and Florida. Plant Foods Hum. Nutr..

[73] Johnson M., Wesely E., Kavitha M., Uma V. (2011). Antibacterial activity of leaves and inter-nodal callus extracts of *Mentha arvensis* L.. Asian Pac. J. Trop. Med..

[74] Mahato D.K., Kargwal R., Kamle M., Sharma B., Pandhi S., Mishra S., Gupta A., Mahmud M.M.C., Gupta M.K., Singha L.B. (2022). Ethnopharmacological properties and Nutraceutical potential of *Moringa oleifera*. Phytomed. Plus.

[75] Behiry S.I., Okla M.K., Alamri S.A., EL-Hefny M., Salem M.Z.M., Alaraidh I.A., Ali H.M., Al-Ghtani S.M., Monroy J.C., Salem A.Z.M. (2019). Antifungal and Antibacterial Activities of *Musa paradisiaca* L. Peel Extract: HPLC Analysis of Phenolic and Flavonoid Contents. Processes.

[76] Shafiei Z., Shuhairi N.N., Yap N.M.F.S., Sibungkil C.-A.H., Latip J. (2012). Antibacterial Activity of *Myristica fragrans* against Oral Pathogens. Evid.-Based Complement. Altern. Med..

[77] Al Abbasy D.W., Pathare N., Al-Sabahi J.N., Khan S.A. (2015). Chemical composition and antibacterial activity of essential oil isolated from Omani basil (*Ocimum basilicum* Linn.). Asian Pac. J. Trop. Dis..

[78] Chimnoi N., Reuk-ngam N., Chuysinuan P., Khlaychan P., Khunnawutmanotham N., Chokchaichamnankit D., Thamniyom W., Klayraung S., Mahidol C., Techasakul S. (2018). Characterization of essential oil from *Ocimum gratissimum* leaves: Antibacterial and mode of action against selected gastroenteritis pathogens. Microb. Pathog..

[79] Sacchetti G., Medici A., Maietti S., Radice M., Muzzoli M., Manfredini S., Braccioli E., Bruni R. (2004). Composition and Functional Properties of the Essential Oil of Amazonian Basil, *Ocimum micranthum* Willd., Labiatae in Comparison with Commercial Essential Oils. J. Agric. Food Chem..

[80] Aruwa C.E., Amoo S.O., Kudanga T. (2019). Extractable and macromolecular antioxidants of *Opuntia ficus*-indica cladodes: Phytochemical profiling, antioxidant and antibacterial activities. S. Afr. J. Bot..

[81] Taïwe G.S., Kuete V., Kuete V. (2017). Chapter 24—Passiflora edulis. Medicinal Spices and Vegetables from Africa.

[82] Mohanasundari C., Natarajan D., Srinivasan K., Umamaheswari S., Ramachandran A. (2007). Antibacterial properties of *Passiflora foetida* L.—A common exotic medicinal plant. Afr. J. Biotechnol..

[83] Slighoua M., Mahdi I., Amrati F.E.-Z., Di Cristo F., Amaghnouje A., Grafov A., Boucetta N., Bari A., Bousta D. (2021). Assessment of in vivo estrogenic and anti-inflammatory activities of the hydro-ethanolic extract and polyphenolic fraction of parsley (*Petroselinum sativum* Hoffm.). J. Ethnopharmacol..

[84] Amarowicz R., Dykes G.A., Pegg R.B. (2008). Antibacterial activity of tannin constituents from *Phaseolus vulgaris*, *Fagoypyrum esculentum*, *Corylus avellana* and *Juglans nigra*. Fitoterapia.

[85] Adedotun I.O., Abdul-Hammed M., Hamzat B.A., Adepoju A.J., Akinboade M.W., Afolabi T.I., Ismail U.T. (2022). Molecular docking, ADMET analysis, and bioactivity studies of phytochemicals from *Phyllanthus niruri* as potential inhibitors of hepatitis C virus NSB5 polymerase. J. Ind. Chem. Soc..

[86] Gurgel A.P.A.D., da Silva J.G., Grangeiro A.R.S., Oliveira D.C., Lima C.M.P., da Silva A.C.P., Oliveira R.A.G., Souza I.A. (2009). In vivo study of the anti-inflammatory and antitumor activities of leaves from *Plectranthus amboinicus* (Lour.) Spreng (Lamiaceae). J. Ethnopharmacol..

[87] Malini S., Kumar S.V., Hariharan R., Bharathi A.P., Devi P.R., Hemananthan E. (2020). Antibacterial, photocatalytic and biosorption activity of chitosan nanocapsules embedded with *Prosopis juliflora* leaf extract synthesized silver nanoparticles. Mater. Today Proc..

[88] Ribeiro P.R., de Castro R.D., Fernandez L.G. (2016). Chemical constituents of the oilseed crop *Ricinus communis* and their pharmacological activities: A review. Ind. Crops Prod..

[89] Amalia A.V., Pukan K.K., Setyawati N., Widiatningrum T., Khasanah U. (2019). Antibacterial activity of Saccharum officinarum leaves extract against food-borne disease. J. Phys. Conf. Ser..

[90] Ajiboye T.O., Mohammed A.O., Bello S.A., Yusuf I.I., Ibitoye O.B., Muritala H.F., Onajobi I.B. (2016). Antibacterial activity of *Syzygium aromaticum* seed: Studies on oxidative stress biomarkers and membrane permeability. Microb. Pathog..

[91] Iseppi R., Sabia C., de Niederhäusern S., Pellati F., Benvenuti S., Tardugno R., Bondi M., Messi P. (2019). Antibacterial activity of *Rosmarinus officinalis* L. and *Thymus vulgaris* L. essential oils and their combination against food-borne pathogens and spoilage bacteria in ready-to-eat vegetables. Nat. Prod. Res..

[92] Weldegebrieal G.K. (2020). Photocatalytic and antibacterial activityof CuO nanoparticles biosynthesized using *Verbascum thapsus* leaves extract. Optik.

[93] Zongo F., Ribuot C., Boumendjel A., Guissou I. (2013). Botany, traditional uses, phytochemistry and pharmacology of *Waltheria indica* L. (syn. Waltheria americana): A review. J. Ethnopharmacol..

[94] (2017). TRAMIL. https://tramil.net/en.

[95] Svobodova B., Barros L., Calhelha R.C., Heleno S., Alves M.J., Walcott S., Bittova M., Kuban V., Ferreira I.C.F.R. (2017). Bioactive properties and phenolic profile of *Momordica charantia* L. medicinal plant growing wild in Trinidad and Tobago. Ind. Crops Prod..

[96] Bendjedid S., Lekmine S., Tadjine A., Djelloul R., Bensouici C. (2021). Analysis of phytochemical constituents, antibacterial, antioxidant, photoprotective activities and cytotoxic effect of leaves extracts and fractions of Aloe vera. Biocatal. Agric. Biotechnol..

[97] Hashemi A.S., Kokab M., Kamalian M., Zarezadeh M., Sheikhpour E., Azod L., Fallah T. (2020). The effect of Aloe vera syrup on prevention of fever and neutropenia in children with acute lymphoid leukemia. Iran.J. Pediatr. Hematol. Oncol..

[98] Paula V.G., Cruz L.L., Sene L.B., Gratão T.B., Soares T.S., Moraes-Souza R.Q., Damasceno D.C., Volpato G.T. (2020). Maternal-fetal repercussions of *Phyllanthus niruri* L. treatment during rat pregnancy. J. Ethnopharmacol..

[99] Gajewski A., Kośmider A., Nowacka A., Puk O., Wiciński M. (2021). Potential of herbal products in prevention and treatment of COVID-19. Literature review. Biomed. Pharmacother..

[100] Fournet J. Flore Illustrée des Phanérogames de Guadeloupe et de Martinique. CIRAD, 2002. Consulté le: 7. https://agritrop.cirad.fr/490202/.

[101] Townsend E.A., Siviski M.E., Zhang Y., Xu C., Hoonjan B., Emala C.W. (2013). Effects of Ginger and Its Constituents on Airway Smooth Muscle Relaxation and Calcium Regulation. Am. J. Respir. Cell Mol. Biol..

[102] Dutta A., Hsiao S.-H., Hung C.-Y., Chang C.-S., Lin Y.-C., Lin C.-Y., Chen T.-C., Huang C.-T. (2023). Effect of [6]-gingerol on viral neuraminidase and hemagglutinin-specific T cell immunity in severe influenza. Phytomed. Plus.

[103] Eddouks M., Ajebli M., Hebi M. (2017). Ethnopharmacological survey of medicinal plants used in Daraa-Tafilalet region (Province of Errachidia), Morocco. J. Ethnopharmacol..

[104] Johji Y., Michihiko M., Rong H.Q., Hisashi M., Hajime F. (1988). The anti-ulcer effect in rats of ginger constituents. J. Ethnopharmacol..

[105] Mascolo N., Jain R., Jain S.C., Capasso F. (1989). Ethnopharmacologic investigation of ginger (*Zingiber officinale*). J. Ethnopharmacol..

[106] Ficker C., Smith M.L., Akpagana K., Gbeassor M., Zhang J., Durst T., Assabgui R., Arnason J.T. (2003). Bioassay-guided isolation and identification of antifungal compounds from ginger. Phytother. Res..

[107] Chen H.C., Chang M.D., Chang T.J. (1985). [Antibacterial properties of some spice plants before and after heat treatment]. Zhonghua Min Guo Wei Sheng Wu Ji Mian Yi Xue Za Zhi.

[108] Horax R., Hettiarachchy N., Kannan A., Chen P. (2010). Proximate composition and amino acid and mineral contents of *Mormordica charantia* L. pericarp and seeds at different maturity stages. Food Chem..

[109] Lawrence R., Tripathi P., Jeyakumar E. (2009). Isolation, Purification and Evaluation of Antibacterial Agents from *Aloe vera*. Braz. J. Microbiol..

[110] Fonseca S.C., Gil L., Manso M.C., Cunha L.M. (2018). Modelling the influence of storage temperature and time after cutting on respiration rate of diced red onions (*Allium cepa* L. cv. Vermelha da Póvoa). Postharvest Biol. Technol..

[111] Ma Y.-L., Zhu D.-Y., Thakur K., Wang C.-H., Wang H., Ren Y.-F., Zhang J.-G., Wei Z.-J. (2018). Antioxidant and antibacterial evaluation of polysaccharides sequentially extracted from onion (*Allium cepa* L.). Int. J. Biol. Macromol..

[112] Rouf R., Uddin S.J., Sarker D.K., Islam M.T., Ali E.S., Shilpi J.A., Nahar L., Tiralongo E., Sarker S.D. (2020). Antiviral potential of garlic (*Allium sativum*) and its organosulfur compounds: A systematic update of pre-clinical and clinical data. Trends Food Sci. Technol..

[113] Yamaguchi Y., Kumagai H. (2020). Characteristics, biosynthesis, decomposition, metabolism and functions of the garlic odour precursor, S-allyl-l-cysteine sulfoxide (Review). Exp. Ther. Med..

[114] Cai L. (1996). Compounds from Syzygium aromaticum Possessing Growth Inhibitory Activity Against Oral Pathogens. J. Nat. Prod..

[115] Costa R., Bisignano C., Filocamo A., Grasso E., Occhiuto F., Spadaro F. (2014). Antimicrobial activity and chemical composition of *Citrus aurantifolia* (Christm.) Swingle essential oil from Italian organic crops. J. Essent. Oil Res..

[116] Atindehou M., Lagnika L., Guérold B., Strub J.M., Zhao M., Van Dorsselaer A., Marchioni E., Prévost G., Haikel Y., Taddéi C. (2013). Isolation and Identification of Two Antibacterial Agents from *Chromolaena odorata* L. Active against Four Diarrheal Strains. Adv. Microbiol..

[117] Nenov N., Gochev V., Girova T., Stoilova I., Atanasova T., Stanchev V., Stoyanova A. (2011). Low Temperature Extraction of Essential Oil Bearing Plants by Liquefied Gases. 6. Barks from Cinnamon (*Cinnamomum zeylanicum* Nees). J. Essent. Oil Bear. Plants.

[118] Kothiwale S.V., Patwardhan V., Gandhi M., Sohoni R., Kumar A. (2014). A comparative study of antiplaque and antigingivitis effects of herbal mouthrinse containing tea tree oil, clove, and basil with commercially available essential oil mouthrinse. J. Indian Soc. Periodontol..

[119] Al-Aamri M.S., Al-Abousi N.M., Al-Jabri S.S., Alam T., Khan S.A. (2018). Chemical composition and in-vitro antioxidant and antimicrobial activity of the essential oil of *Citrus aurantifolia* L. leaves grown in Eastern Oman. J. Taibah Univ. Med. Sci..

[120] Erel Ş., Reznicek G., Şenol S., Yavaşoğlu N., Konyalioğlu S., Zeybek A. (2012). Antimicrobial and antioxidant properties of *Artemisia* L. species from western Anatolia. Turk. J. Biol..

[121] Pandey B.P., Thapa R., Upreti A. (2017). Chemical composition, antioxidant and antibacterial activities of essential oil and methanol extract of *Artemisia vulgaris* and *Gaultheria fragrantissima* collected from Nepal. Asian Pac. J. Trop. Med..

[122] Ene A.C., Atawodi S.E., Fatihu M.Y. (2014). Acute Toxicity of Chloroform Extract of *Artemisia macivera* Linn in Swiss Albino Mice. J. Pharm. Res. Int..

[123] Radulović N.S., Genčić M.S., Stojanović N.M., Randjelović P.J., Stojanović-Radić Z.Z., Stojiljković N.I. (2017). Toxic essential oils. Part V: Behaviour modulating and toxic properties of thujones and thujone-containing essential oils of *Salvia officinalis* L., *Artemisia absinthium* L., *Thuja occidentalis* L. and *Tanacetum vulgare* L.. Food Chem. Toxicol..

[124] Ibrahim D., Hong L.S., Kuppan N. (2013). Antimicrobial Activity of Crude Methanolic Extract from *Phyllanthus niruri*. Nat. Prod. Commun..

[125] El-Saber Batiha G., Beshbishy A.M., Wasef L.G., Elewa Y.H.A., Al-Sagan A.A., El-Hack M.E.A., Taha A.E., Abd-Elhakim Y.M., Devkota H.P. (2020). Chemical Constituents and Pharmacological Activities of Garlic (*Allium sativum* L.): A Review. Nutrients.

[126] Khaerunnisa S., Kurniawan H., Awaluddin R., Suhartati S., Soetjipto S. (2020). Potential Inhibitor of COVID-19 Main Protease (Mpro) From Several Medicinal Plant Compounds by Molecular Docking Study. Preprints.

[127] Di Pierro F., Khan A., Iqtadar S., Mumtaz S.U., Chaudhry M.N.A., Bertuccioli A., Derosa G., Maffioli P., Togni S., Riva A. (2023). Quercetin as a possible complementary agent for early-stage COVID-19: Concluding results of a randomized clinical trial. Front. Pharmacol..

[128] Cunningham A.C., Goh H.P., Koh D. (2020). Treatment of COVID-19: Old tricks for new challenges. Crit. Care Lond. Engl..

[129] Fitero A., Bungau S.G., Tit D.M., Endres L., Khan S.A., Bungau A.F., Romanul I., Vesa C.M., Radu A.-F., Tarce A.G. (2022). Comorbidities, Associated Diseases, and Risk Assessment in COVID-19—A Systematic Review. Int. J. Clin. Pract..

[130] Kabir M.T., Uddin M.S., Hossain M.F., Abdulhakim J.A., Alam M.A., Ashraf G.M., Bungau S.G., Bin-Jumah M.N., Abdel-Daim M.M., Aleya L. (2020). nCOVID-19 Pandemic: From Molecular Pathogenesis to Potential Investigational Therapeutics. Front. Cell Dev. Biol..

[131] Arnab R., Arnab R. (2017). Chapter 7—Stratified Sampling. Survey Sampling Theory and Applications.

[132] Sreekeesoon D.P., Mahomoodally M.F. (2014). Ethnopharmacological analysis of medicinal plants and animals used in the treatment and management of pain in Mauritius. J. Ethnopharmacol..

[133] Cadena-González A.L., Sørensen M., Theilade I. (2013). Use and valuation of native and introduced medicinal plant species in Campo Hermoso and Zetaquira, Boyacá, Colombia. J. Ethnobiol. Ethnomed..

[134] Siddique H., Pendry B., Rashid M.A., Rahman M.M. (2021). Medicinal plants used to treat infectious diseases in the central part and a northern district of Bangladesh—An ethnopharmacological perception. J. Herb. Med..

[135] Friedman J., Yaniv Z., Dafni A., Palewitch D. (1986). A preliminary classification of the healing potential of medicinal plants, based on a rational analysis of an ethnopharmacological field survey among Bedouins in the Negev Desert, Israel. J. Ethnopharmacol..

